# Investigation of Macroscopic Mechanical Behavior of Magnetorheological Elastomers under Shear Deformation Using Microscale Representative Volume Element Approach

**DOI:** 10.3390/polym16101374

**Published:** 2024-05-11

**Authors:** Ilda Abdollahi, Ramin Sedaghati

**Affiliations:** Department of Mechanical, Industrial and Aerospace Engineering, Concordia University, Montreal, QC H3G 1M8, Canada

**Keywords:** magnetorheological elastomers (MREs), representative volume element (RVE), periodic boundary condition (PBC), finite element method, hyper-elastic materials

## Abstract

Magnetorheological elastomers (MREs) are a class of smart materials with rubber-like qualities, demonstrating revertible magnetic field-dependent viscoelastic properties, which makes them an ideal candidate for development of the next generation of adaptive vibration absorbers. This research study aims at the development of a finite element model using microscale representative volume element (RVE) approach to predict the field-dependent shear behavior of MREs. MREs with different elastomeric matrices, including silicone rubber Ecoflex 30 and Ecoflex 50, and carbonyl iron particles (CIPs) have been considered as magnetic particles. The stress–strain characteristic of the pure silicon rubbers was evaluated experimentally to formulate the nonlinear Ogden strain energy function to describe hyper-elastic behavior of the rubbery matrix. The obtained mechanical and magnetic properties of the matrix and inclusions were integrated into COMSOL Multiphysics to develop the RVE for the MREs, in 2D and 3D configurations, with CIP volume fraction varying from 5% to 40%. Periodic boundary condition (PBC) was imposed on the RVE boundaries, while undergoing shear deformation subjected to magnetic flux densities of 0–0.4 T. Comparing the results from 2D and 3D modeling of isotropic MRE-RVE with the experimental results from the literature suggests that the 3D MRE-RVE can be effectively used to accurately predict the influence of varying factors including matrix type, volume fraction of magnetic particles, and applied magnetic field on the mechanical behavior of MREs.

## 1. Introduction

Magnetorheological (MR) materials are a class of smart materials with the unique ability to change their physical or mechanical characteristics rapidly in less than few milliseconds in response to an external magnetic field. These materials are fabricated by dispersing magnetic particles inside nonmagnetic host matrices. MR fluid is a commonly used MR material which comes with some drawbacks, such as magnetic particle sedimentation, sealing problems, and environmental contamination [1,2,3,4,5]. These challenges are not encountered by MR elastomers (MREs), because the magnetic particles are often bonded by a carrier matrix, like rubber. MREs stand as multi-functional materials, exhibiting the capability of dynamically altering their mechanical properties, including stiffness, and damping capacity, in response to an external magnetic field. This characteristic is measured by defining magnetorheological (MR) effect as the ratio of the change in the field-dependent physical or mechanical property to the value of the same property when no field is applied [6,7,8].

MREs comprise three fundamental components: magnetic particles, nonmagnetic elastic matrices, and additives [9,10]. Due to the application of a magnetic field during the curing process, it is possible to fabricate MREs with an anisotropic particle-formed microstructure, and when no field is applied during the curing process, prepared MREs have an isotropic particle-formed microstructure [11,12]. MREs show great potential to be incorporated into the design of intelligent devices in a variety of engineering disciplines [13,14,15].

Jolly et al. [16] has pioneered the investigation on the mechanical response of anisotropic elastomer composites with embedded CIP under the application of magnetic fields, revealing significant changes in shear modulus of MREs in response to the magnetic field. Davis [17] proposed a phenomenological model to predict the shear modulus of isotropic and anisotropic MREs, with and without the application of the external magnetic field. His study suggested that magnetic particles with 27% volume fraction is an optimal content with respect to MR effect. Berasategi et al. [18] studied silicone-based isotropic and anisotropic MREs containing CIP concentrations ranging from 5% to 30% volume content, indicating changes in storage modulus and loss modulus as CIP content increased. Vatandoost et al. [19] investigated pre-strain effects on compression mode dynamic characteristics of isotropic and anisotropic MREs. Their results revealed that pre-strain has a significantly nonlinear impact on the elastic and loss moduli. Syam et al. [20] conducted a finite element analysis on MREs’ behavior at the microscale, using the COMSOL software (v. 5.4). However, they used linear material model for silicone rubber as the matrix material. Their results showed increased stiffness in both linear and torsional modes under the application of an external magnetic field. Asadi Khanouki et al. [21] examined isotropic and anisotropic silicone rubber based MREs with various contents of CIP, and proposed a microscale modeling that was validated with the experimental results. Dargahi et al. [22] fabricated different MRE samples in terms of rubber matrix and ferromagnetic particle contents and conducted static and dynamic shear tests on the samples. Their results showed a significant 1672% increase in storage modulus under 0.45 T magnetic flux density.

Sun et al. [23] conducted a finite element analysis on the shear deformation of isotropic MREs under the application of an external magnetic field, using the concept of representative volume element (RVE) in 2D configuration, in COMSOL. However, they approximated the nonlinear B-H properties of magnetic particles using a linear model and assumed the relative permeability of CIP to be 100. Inspired by their work, Xu et al. [24] conducted the 3D modeling of isotropic MREs in tensile mode under the application of a magnetic field in COMSOL. They used a linear material model to describe the nonlinear hyper-elastic behavior of the host elastomeric matrix, and the linear magnetic model was used to describe the magnetic properties of CIP, thus ignoring the saturation. Kiarie et al. [25] adopted a 2D RVE approach using COMSOL to predict the magnetic field-induced strain in MREs, and they employed the Mooney–Rivlin nonlinear material model for the host rubber; however, their problem did not include any mechanical load or displacement imposed on the RVE. Li et al. [26] studied the magnetic field-induced shear behavior of MREs using a 2D RVE approach in COMSOL for both isotropic and anisotropic MREs; however, they assumed the magnetic particles (hydroxy iron powder) to have a very high relative permeability of 5000, and the silicone rubber behavior to be like a linear elastic matrix.

Hence, due to the prohibitive and expensive experimental procedures to characterize the behavior of MREs under the influence of various factors, the development of a promising analytical model to predict the mechanical properties of MREs offers several benefits over experiments, including controlled simulations, broader scenario exploration, cost-effectiveness, and environmental sustainability. To the best of the authors’ knowledge and by reviewing the literature, there is a gap in the literature concerning taking the nonlinear behavior of MRE components into account in FE modeling process, and studies so far have faced issues in taking this inherent nonlinearity into account. Therefore, in this research study, the representative volume element (RVE) approach as an FE modeling scheme was effectively utilized to model the shear deformation of MREs under the influence of an external magnetic field, considering material and magnetic nonlinearities into account. An appropriate RVE size was defined for modeling the MRE, and periodic boundary condition (PBC) was imposed on the RVE boundaries. Experiments were then carried out on host rubber samples (silicone rubber) to obtain the stress–strain data. The extracted data was used to formulate the Ogden strain energy to describe the nonlinear hyper-elastic behavior of the rubber material. The other nonlinearity in the MRE is attributed to the magnetic behavior of CIP, which was described through a B-H curve, considering the saturation, instead of using a high relative permeability as used in previous studies.

The RVE was then generated in COMSOL Multiphysics (v. 6.0) in 2D and 3D configurations, and pure shear deformation was incrementally applied on the RVE, while PBC was imposed on the RVE boundaries. Simultaneously, a homogeneous magnetic field was created in the surrounding air domain perpendicular to the shear direction, and the Maxwell stress tensor was defined on the CIP inclusion. The influence of different factors like CIP volume fraction, magnetic field intensity, and host rubber’s mechanical behavior were investigated on the shear behavior of the MRE. The results revealed the significant credibility of the developed 3D model in predicting the MREs’ shear behavior, exhibiting a substantial potential to be used for the prediction of MREs’ mechanical behavior as a reliable alternative to costly experiments.

## 2. Materials and Methods

### 2.1. Representative Volume Element (RVE)

A fundamental goal in the field of heterogeneous materials physics is to determine the effective mechanical properties of these materials. Among the proposed techniques, representative volume element (RVE) homogenization stands out as a method that utilizes a statistically homogeneous representation of heterogeneous materials at a microscale to derive their effective properties on a macroscale. Many researchers have suggested different aspects to be considered in the evaluation of the RVE size; however, these definitions share a core idea that RVE is defined as the minimal volume element when compared to the macroscopic dimensions of the structure, yet large enough to include a substantial amount of information about the microstructure, that exhibits an equivalent target attribute or behavior to that of the whole material at a macroscopic scale [27,28,29,30].

### 2.2. The RVE Size for Models Containing Hard Particles

To obtain an accurate estimation of the effective properties, it is essential to establish a correlation between the size of the RVE and the various morphological, mechanical, and thermal factors associated with microstructures [31,32]. El Moumen et al. [30] adopted a combined numerical–statistical approach in order to investigate the variation in the RVE size with respect to different parameters in the microstructure. The geostatistical parameter, integral range “*A*” in the context of composite materials, was used to relate the size of the RVE with other microstructure parameters and was defined by previous researchers [33,34,35]. The integral range for random microstructures with a volume fraction ϕ may be expressed as follows [32]:

(1) $$A=\frac{\phi }{V_{RVE}}$$

For a stationary random function *z*, the variance of *z*, $D_{z}^{2}\left(V\right)$, over the volume *V* is attainable as a function of the integral range *A* and the point variance $S_{z}^{2}$ [30] as follows:

(2) $$D_{z}^{2}\left(V\right)=S_{z}^{2}\;\frac{A}{V}$$

Considering a stochastic microstructure composed of two distinct phases, denoted as $F_{1}$ with its distinct real characteristics, namely, $z_{1}$ and $F_{2}$ with characteristics $z_{2}$, where phase $F_{1}$ occupies a volume percentage of ϕ, and phase $F_{2}$ occupies a volume fraction of (1 – ϕ), the point variance $S_{z}^{2}$ of the random variable *z* in the context of a two-phase material may be provided as follows [30]:

(3) $$S_{z}^{2}=\;\phi \;\left(1-\phi \right){\left(z_{1}-z_{2}\right)}^{2}$$

Given the current context, whereby the mechanical properties are represented as the random variable *z*, substituting Equation (3) in Equation (2), the volume variance,$\;D_{z}^{2}\left(V\right)$, can be evaluated as follows:

(4) $$D_{z}^{2}\left(V\right)=\;\phi \;\left(1-\phi \right){\left(z_{1}-z_{2}\right)}^{2}\;\frac{A}{V}$$

where $D_{z}^{2}\left(V\right)$ is the variance of the volume *V*, and *A* is the integral range. To determine the RVE parameters, the number of realizations *n* and the absolute error $\varepsilon_{abs}$ were used to express $D_{z}^{2}\left(V\right)$ as follows [26]:

(5) $$4D_{z}^{2}\left(V\right)={\varepsilon_{abs}}^{2}\;n$$

It should be noted that the determination of the size of the RVE involves defining the volume at which the number of realizations is equivalent to one [36]. Therefore, using Equations (1), (4), and (5) and considering $n\left(V=V_{RVE}\right)=1$, we can write the following:

(6) $$V_{RVE}=\frac{4\;\phi^{2}\;\left(1-\phi \right){\left(z_{1}-z_{2}\right)}^{2}\;}{\begin{array}{c} {\varepsilon_{abs}}^{2}\;V_{RVE}\; \end{array}}$$

Defining the contrast ratio (c) in mechanical characteristics as c=z2z1, in which $z_{2}$ represents the matrix property (phase $F_{2})$ and $z_{1}$ corresponds to the property of phase $F_{1}$, the representativity of the estimated characteristics in random microstructures may be determined by considering the volume size, with respect to the desired relative error εr=εabsz2. Hence, using Equation (6), the final expression for RVE size would be the following:

(7) $$V_{RVE}\left(c,\phi \right)=\frac{2\left|1-c\right|\phi \;\sqrt{\left(1-\phi \right)}}{\varepsilon_{r}}$$

Equation (7) provides a clear relationship between the representative volume element (RVE) of random microstructures and both the volume fraction and contrast ratio, while also accounting for a desirable and fixed relative error.

For MREs, the two phases include soft elastomeric phase ($F_{2}$) impregnated with hard solid spherical inclusions, phase ($F_{1}$), represented by the micron-sized carbonyl iron powder (CIP). Thus, for MREs, the contrast ratio *c* which here represents the ratio of the modulus of the rubbery matrix to that of solid inclusions is nearly negligible compared with unity. Therefore, using Equation (7) and assuming c=0, the RVE size can be determined for different particle volume fractions and the desired relative error. Tuning the aforementioned parameters to a negligible relative error, the equation leads us to an RVE with one CIP inclusion in a cubic rubber matrix, which is the same RVE size presented by Davis [17].

### 2.3. Periodic Boundary Conditions

The commonly employed boundary conditions in micromechanics fall into two categories of uniform boundary conditions: Dirichlet, also known as Displacement Boundary Condition (DBC), and Neumann, also known as Traction Boundary Condition (TBC). However, TBC tends to overestimate the effective material properties, while DBC underestimates them. Furthermore, researchers have also developed periodic boundary conditions (PBC) which are typically applied to unit cells in cases where the heterogeneous material exhibits a periodic structure [37,38,39]. It has been reported that PBC yields more precise effective modulus estimates compared with other conventional boundary conditions and is less sensitive to the RVE size or inclusion position in the unit cell [40]. In the following, PBC in 2D and 3D configurations are briefly discussed.

#### 2.3.1. Periodic Boundary Conditions in 2D

Considering the periodic structure of the macroscopic body and a square unit cell, for each boundary pair, compatibility is essential in line with the periodicity assumption. This implies that the deformation of each boundary pair is identical, and the stress vectors have opposite signs on each pair [37,38]. Smit et al. [41] derived the suitable displacement boundary conditions as follows:

(8) $$u_{12}-u_{v_{4}}=u_{11}-u_{v_{1}}\;u_{22}-u_{v_{2}}=u_{21}-u_{v_{2}}\;u_{v_{3}}-u_{v_{2}}=u_{v_{4}}-u_{v_{1}}$$

where $u_{ij}$ denotes the displacement vector associated with any material point situated on the corresponding boundary $\Gamma_{ij}$, while $u_{v_{i}}$ represents the displacement vector attributed to each vertex $v_{i}$. To eliminate rigid body motions, it is necessary to impose $u_{v_{k}}=0$ for any *k* within the set $k\in \left\{1,2,4\right\}$.

The micro–macro relations for the total stress and strain tensors can be established as follows:

(9) $$\sigma_{ij\;\left(macro\right)}=\langle \sigma_{ij}\rangle ,\;\varepsilon_{ij\;\left(macro\right)}=\langle \varepsilon_{ij}\rangle$$

where $\sigma_{ij\;\left(macro\right)}\;$ and $\varepsilon_{ij\;\left(macro\right)}\;$signify the macroscopic total stress and strain tensors, respectively, while $\langle \sigma_{ij}\rangle$ and $\langle \varepsilon_{ij}\rangle$ represent the corresponding microscopic averages over the surface of the unit cell (i.e., $\langle \sigma_{ij}\rangle =\frac{1}{S}\int_{R}^{\;}\sigma_{ij}\;dy$; $S=\int_{R}^{\;}\;dy$).

Utilizing the averaged elastic constitutive equations in Equation (9), the expressions for the affective elastic properties can be obtained as follows:

(10) $$E_{11}^{eff}=\frac{\sigma_{11\;\left(macro\right)}\;}{\varepsilon_{11\;\left(macro\right)}}\;\;,\;\nu_{12}^{eff}=-\frac{\varepsilon_{22\;\left(macro\right)}\;}{\varepsilon_{11\;\left(macro\right)}}$$

where $E_{11}^{eff}$ is the effective Young’s modulus, and $\nu_{12}^{eff}$ denotes the effective Poisson’s ratio.

#### 2.3.2. Periodic Boundary Conditions in 3D

A 3D RVE featuring a periodic microstructure can be considered as a cubic structure which contains both fiber and matrix constituents. This cube is bounded by six surfaces, ensuring that any two parallel surfaces always maintain parallel alignment along either the x-, y-, or z-axes. Each two parallel surfaces are distinguished by assigning an index (*POS/NEG*) based on their location on the associated coordinate axis. For example, assuming a cubic RVE with the dimension denoted as *D*, the $X_{POS}$ surface corresponds to the y–z plane situated at the maximum x-axis cubic dimension (i.e., x = *D*), while the $X_{NEG}$ surface is located at the minimum x-axis cubic dimension (i.e., x = 0).

Each of these surfaces consists of nodes, and nodes located on the $X_{POS}$ surface are referred to as $X_{POSnodes}$. Similar terminology applies to the other five faces. The set of nodes for a specific surface is defined as $S_{np}$, where *n* represents the reference frames (X, Y, Z), and *p* encompasses either the positive or negative faces along a given axis. Consequently, similar to the concept of PBC in 2D configuration, the mathematical expressions governing the imposition of periodic deformation on all nodes in the three dimensions of the 3D RVE domain can be mathematically described as follows [42]:

$$U_{i}^{S_{XPOS}}-U_{i}^{S_{XNEG}}-U^{N_{2}}+U^{N_{1}}=0\;$$

(11) $$U_{i}^{S_{YPOS}}-U_{i}^{S_{YNEG}}-U^{N_{3}}+U^{N_{1}}=0\;$$

$$U_{i}^{S_{ZPOS}}-U_{i}^{S_{ZNEG}}-U^{N_{4}}+U^{N_{1}}=0\;$$

Enforcing periodic boundary conditions requires stress equilibrium across opposite surfaces within the RVE domain. For every surface $S_{np}$ in the 3D RVE, a specific unit outward normal vector is defined as $n_{np}$. Assuming that the domain is experiencing stress, the condition for stress equilibrium across opposing pairs of surfaces is achieved in the following situations:

$$\sigma \;n_{XPOS\;}\left(y,z\right)=-\sigma \;n_{XNEG\;}\left(y,z\right)$$

(12) $$\sigma \;n_{YPOS\;}\left(x,z\right)=-\sigma \;n_{YNEG\;}\left(x,z\right)$$

$$\sigma \;n_{ZPOS\;}\left(x,y\right)=-\sigma \;n_{ZNEG\;}\left(x,y\right)$$

where σ is the stress tensor.

Ultimately, the volume-averaged stress within the periodically deformed RVE domain can be expressed as follows:

(13) $$\langle \sigma \rangle =\frac{1}{V}\int_{V}^{\;}\sigma \;dV$$

where *V* is the RVE volume, and the volume-averaged stress is denoted as $\langle \sigma \rangle$. Under the assumption of global periodicity within the RVE domain, the overall macroscopic stress and the global strain are expressed as $\langle \sigma \rangle =\sigma_{macro}$ and $\varepsilon_{macro}\;$ respectively. $\varepsilon_{macro}$ is determined based on the displacements of the retained nodes, which are calculated as follows:

(14) $$u_{2}=\varepsilon_{macro}\left(X_{2}-X_{1}\right),\;\;u_{3}=\varepsilon_{macro}\left(X_{3}-X_{1}\right),\;\;\;\;\;u_{4}=\varepsilon_{macro}\left(X_{4}-X_{1}\right)$$

Here, $u_{i}$ represents the displacement vector of retained node, *i*, in relation to its coordinate position, $X_{i}$, where $u_{i}$ = [$u_{i,x}$, $u_{i,y}$, $u_{i,z}$]. It should be noted that $u_{1}$ is restricted to be zero to avoid rigid body motion.

The effective properties could be evaluated consequently in the same fashion as explained in 2D. For example, the following are the effective shear moduli obtained from simple shear deformation along XY, XZ, and YZ planes, respectively.

(15) $$G_{12}^{eff}=\frac{\tau_{12\;\left(macro\right)}\;}{\gamma_{12\;\left(macro\right)}}\;\;,\;\;G_{13}^{eff}=\frac{\tau_{13\;\left(macro\right)}\;}{\gamma_{13\;\left(macro\right)}}\;\;\;,\;\;G_{23}^{eff}=\frac{\tau_{23\;\left(macro\right)}\;}{\gamma_{23\;\left(macro\right)}}\;$$

where τ  and γ are shear stress and shear strain, respectively.

### 2.4. Maxwell’s Stress Tensor

The force acting on a point charge *q* moving with velocity *v* in the presence of both an electric field *E* and a magnetic field *B* is described by the Lorentz force equation which is a fundamental concept in electromagnetism and is expressed as follows [43]:

(16) $$F=q\left(E+v\times B\right)$$

Using the Maxwell equations in electromagnetism and the Lorentz force, we can introduce Maxwell Stress tensor as follows [43]:

(17) $$T_{ij\;}=\epsilon_{0}(E_{i\;}E_{j\;}-\frac{1}{2}\delta_{ij\;}E^{2})+\frac{1}{\mu_{0}}\;(B_{i\;}B_{j\;}-\frac{1}{2}\delta_{ij\;}B^{2})$$

where $\mu_{0}$ and $\epsilon_{0}$ denote vacuum permeability and permittivity, respectively, and$\;\delta_{ij\;}\;$is the Kronecker delta which is defined as follows:

(18) $$\left\{\begin{array}{c} \delta_{ij\;}=1\;\;\;\;\;\;\;\;;i=j\\ \delta_{ij\;}=0\;\;\;\;\;\;\;\;;i\neq j \end{array}\right.\;$$

In this study, MREs are only exposed to uniform magnetic fields; thus, the first part of Equation (17), containing the electric field, is eliminated, yielding the Maxwell stress tensor as follows:

(19) $$T_{ij\;}=\frac{1}{\mu_{0}}\;(B_{i\;}B_{j\;}-\frac{1}{2}\delta_{ij\;}B^{2})$$

### 2.5. Governing Equations

Considering the basic balance principles of continuum mechanics, including the linear momentum and angular momentum balance principles, there exists the equation of mechanical equilibrium and is as follows [44]:

(20) $$\nabla \cdot \sigma +\rho f=\rho \dot{v}$$

Here, *σ* represents the stress tensor, *ρ* is density, *f* represents the body forces, and *v* is the velocity. In the case of static or quasi static conditions ($\dot{v}$ = 0), the force balance equation simplifies to the following:

(21) ∇·σ+ρf=0

When coupling magnetic and elastic behavior, different methods can be used to define body forces and stresses. The deformation of the material due to a magnetic field can be incorporated into the force balance equation in terms of the body force represented by the magnetic force per unit volume, denoted as $f_{m}$. Assuming that the body force due to the weight is negligible, Equation (21) can be described as follows:

(22) $$\nabla \cdot \sigma +\rho \;\left(f_{m}\right)=0$$

Alternatively, Equation (22) can be expressed in terms of the total stress tensor ***T*** as follows:

(23)     ∇·T=0  ;          T=σ+T 

Here, ***T*** contributes to the sum of mechanical and magnetic stress tensors as T=σ+T, where T  is defined in Equation (19).

### 2.6. Characterization of Elastomeric Matrix and Magnetic Inclusion in MREs

**Figure 1:**
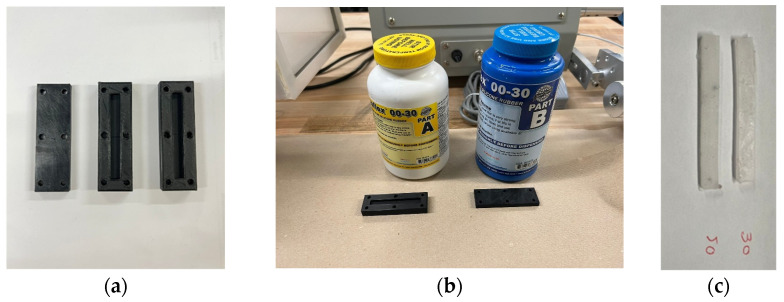
(**a**) The identical molds fabricated by the 3D printer, (**b**) parts A and B for fabricating silicone rubber Ecoflex 30 to be mixed and cured, and (**c**) the cured final samples (30 indicates Ecoflex 30, and 50 refers to Ecoflex 50).

In order to generate the RVE for predicting the shear modulus of MRE, two datasets are required: the mechanical and magnetic properties of the pure rubber material (the matrix) and carbonyl iron particle (CIP) (the ferromagnetic inclusions). As for the rubber material, silicone rubber was chosen and produced in the laboratory using two different types of silicone rubber, represented as Ecoflex 30 and Ecoflex 50 (Smooth-On, Macungie, PA, USA). In order to fabricate identical samples in terms of shape and dimensions, two rectangular molds of 37 × 6 × 3 [mm] were fabricated using a 3D-printer (Original Prusa i3 MK3S+, Prusa Research, Prague, Czech Republic), as shown in Figure 1a.

To fabricate the silicone rubber samples, platinum-based silicone rubber from Smooth-On, Macungie, PA, USA was used, comprising two parts to be thoroughly mixed and cured. The two parts denoted as the rubber part (A) and the catalyst part (B), as shown in Figure 1b, were added, and stirred in a 50–50 weight fraction. The primary mixture was then placed in the conditioning vacuum mixer (THINKY: ARV-200, THINKY CORPORATION, Laguna Hills, CA, USA) for 40 s under 2000 rpm to be thoroughly mixed and degassed. The final mixture was then poured into the molds and cured at room temperature for 15 h. Finally, the vulcanized samples were removed from the molds and were ready for conducting the tensile test. Figure 1c shows the fabricated samples.

#### 2.6.1. Characterization of the MRE’s Elastomeric Matrix Using the Uniaxial Tensile Test

**Figure 2:**
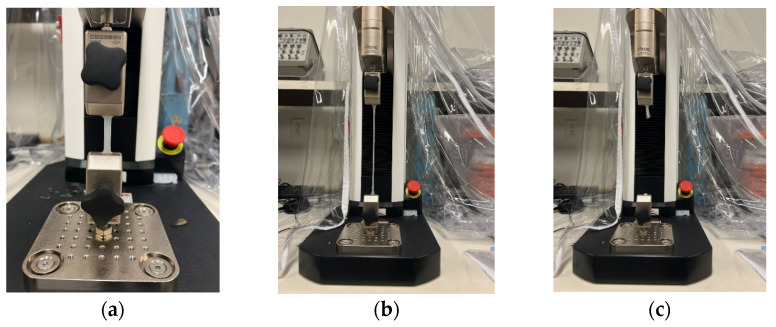
(**a**) Silicone rubber sample (Ecoflex 30) assembled on the MTS machine prior to the tensile test, (**b**) sample after the final steps of tensile test, and (**c**) the failed sample.

**Figure 3:**
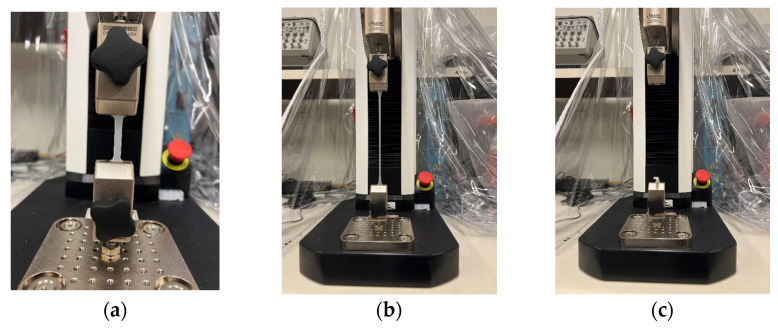
(**a**) Silicone rubber sample (Ecoflex 50) assembled on the MTS machine prior to the tensile test, (**b**) sample after the final steps of tensile test, and (**c**) the failed sample.

In pursuit of determining the viscoelastic properties of silicone rubber samples, the two identical rectangular samples with dimensions of 37 × 6 × 3 [mm] underwent pure tensile to failure test with 30 [mm/min] velocity, using an MTS machine (F1505-IM, Mark–10, Copiague, NY, USA), under identical conditions. Figure 2 and Figure 3 illustrate the three significant steps of the conducted test for silicone rubber Ecoflex 30 and Ecoflex 50, respectively.

**Figure 4:**
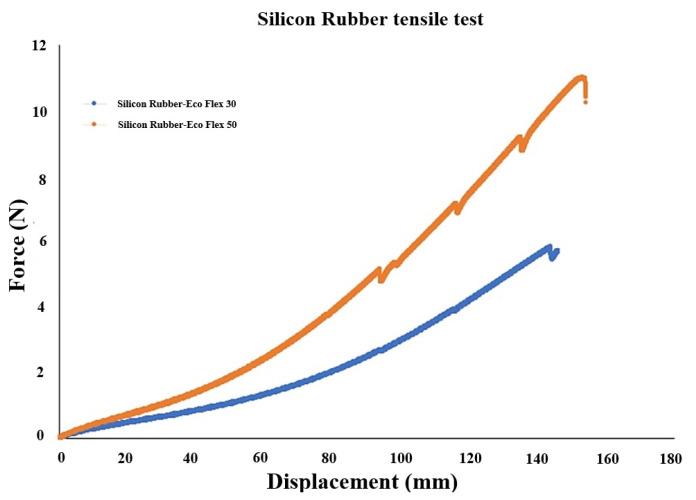
The extracted raw data of the conducted pure tensile to failure test for silicone rubber-Ecoflex 30 and Ecoflex 50.

The extracted force–displacement experimental data for silicone rubber Ecoflex 30 and Ecoflex 50 are shown and compared in Figure 4. For the given force, silicone rubber Ecoflex 30 experiences larger displacement compared with Ecoflex 50 due to its lower stiffness. The force–displacement data were then used to obtain stress–stretch data that were subsequently utilized to identify the material parameters of hyper-elastic Ogden material model [45]. Compared with other hyper-elastic models such as Neo-Hookean and Mooney–Rivlin, the Ogden model was shown to provide the better prediction [45,46].

To characterize the behavior of the rubber material, the strain energy function in the theory of hyper-elasticity, which represents the stored energy in the material during the deformation and is denoted as *W*, is employed. This energy function is dependent on the principal stretches (*λ_1_*, *λ*_2_, and *λ*_3_) which are stretch ratios defined as deformed length divided by the original length for the unit fibers oriented along the principal directions [47,48]. Taking into consideration that rubber materials can be generally considered incompressible, we have $\lambda_{1}\lambda_{2}\;\lambda_{3}=1.\;$The principal Cauchy stresses, denoted as $\sigma_{i}$ (*i* = 1, 2, 3), are intricately connected to the stretches through the derivative of the strain energy function, as expressed by the following equation [46]:

(24) $$\sigma_{i}=\lambda_{i}\;\frac{\partial W}{\partial \lambda_{i}}-L$$

Here, the index i does not represent a dummy index, and there is no summation over it, and *L* serves as an unknown Lagrange multiplier, associated with the aforementioned incompressibility constraint. For the case of pure tension uniaxial tensile test, we have $\sigma_{2}$ = $\sigma_{3}$ = 0, and hence, by expressing the following equation and using Equation (24), we can effectively eliminate the unknown Lagrange multiplier *L.*

(25) $$\left(\sigma_{1}-\sigma_{2}\right)=\left(\sigma_{1}-\sigma_{3}\right)=\sigma_{1}$$

The Ogden strain energy function can accurately describe the nonlinear behavior of hyper-elastic materials, and for incompressible materials, it takes the following form [46]:

(26) $$W=\overset{N}{\underset{p=1}{\sum }}\frac{\mu_{p}}{\alpha_{p}}\;\left({\lambda_{1}}^{\alpha_{p}}+{\lambda_{2}}^{\alpha_{p}}+{\lambda_{3}}^{\alpha_{p}}-3\right)\;\;\;$$

Here, each $\mu_{p}$ and $\alpha_{p}$ represents material characteristic parameters, to be determined using experimental data. For practical application, the summation in Equation (26) is confined to a finite number of terms. However, to maintain consistency with classical theory of incompressible isotropic elasticity, these constant parameters must adhere to the following condition:

(27) $$\overset{N}{\underset{p=1}{\sum }}\mu_{p}\;\alpha_{p}=2G\;\;\;$$

where *N* is a positive integer, and G is the shear modulus of the material in its undeformed stress-free (natural) configuration, which implies that $\overset{N}{\underset{p=1}{\sum }}\mu_{p}\;\alpha_{p}>0.$ In the present research study, the three-term Ogden model (*N* = 3) was adopted due to its better accuracy compared to one-term and two-term Ogden model [45]. Using the three-term Ogden strain energy function in Equation (26), and considering Equations (24) and (25), the principal value of the Cauchy stress can be obtained as follows:

(28) $$\sigma =\overset{3}{\underset{p=1}{\sum }}\mu_{p}\left({\lambda_{i}}^{\alpha_{p}-1}-{\lambda_{i}}^{-(\frac{1}{2}\alpha_{p}+1)}\right)$$

The extracted data from the pure tensile test was then employed to determine the material parameters in Ogden strain-energy function through least squares (LS) optimization technique. Let us consider a vector Λ = [$\Lambda_{1},\;\Lambda_{2},\;\ldots ,\;\Lambda_{m}$]*ᵀ*, representing a collection of experimental deformation values, and associated vector *S =* $[S_{1},\;S_{2},\;\ldots ,\;S_{m}$]*ᵀ*, corresponding to stress values, in which *m* represents the number of datasets.

For the given deformation vector *Λ*, using Ogden material model, the principal Cauchy stress in Equation (28) can be expressed as *σ* ($\mu_{p}$, $\alpha_{p}$), in which the material parameters $\mu_{p}$ and $\alpha_{p}$ are unknown. It is noted that, for the three-term Ogden model, *p* = 1 to 3, and thus, the number of unknown material parameters are 6 (i.e., $\mu_{1}$, $\mu_{2}$,$\;\mu_{3}$, $\alpha_{1},$ $\alpha_{2},\;\alpha_{3}$). A least square minimization problem has been subsequently formulated to identify the material parameters in order to minimize the error between experimental and model results. The error function may thus be defined as follows:

(29) $$E_{r}=\overset{m}{\underset{j=1}{\sum }}{\left[1-\frac{\;\sigma \left(\mu_{p}\;,\;\alpha_{p}\right)}{S_{j}}\;\right]}^{2}\;$$

Now, considering Equation (28), the optimization problem can be formulated as follows:

Find the design variables: $\left\{\mu_{1},\;\mu_{2},\;\mu_{3},\alpha_{1},\;\alpha_{2},\;\alpha_{3}\right\}$

To minimize $E_{r}$;

(30) $$E_{r}=\overset{m}{\underset{j=1}{\sum }}{\left[1-\frac{\sum_{p=1}^{3}\mu_{p}\left({\lambda_{j}}^{\alpha_{p}-1}-{\lambda_{j}}^{-(\frac{1}{2}\alpha_{p}+1)}\right)}{S_{j}}\;\right]}^{2}$$

Subjected to: $\overset{3}{\underset{p=1\;}{\sum }}(-\mu_{p}\alpha_{p}\;)<0$.

The optimization problem in Equation (30) has been solved using stochastic-based Genetic Algorithm (GA) and hybrid method based on the combination of GA and gradient-based Sequential Quadratic Programming (SQP) method. In the hybrid method, the optimal solution from GA has been fed into the SQP as the initial point in an attempt to accurately identify the global optimum solution. The identified optimal Ogden material parameters using GA and GA + SQP for both Ecoflex 30 and Ecoflex 50 silicone rubbers are provided in Table 1. The basic mechanical and magnetic properties of silicone rubber are also presented in Table 2.

**Figure 5:**
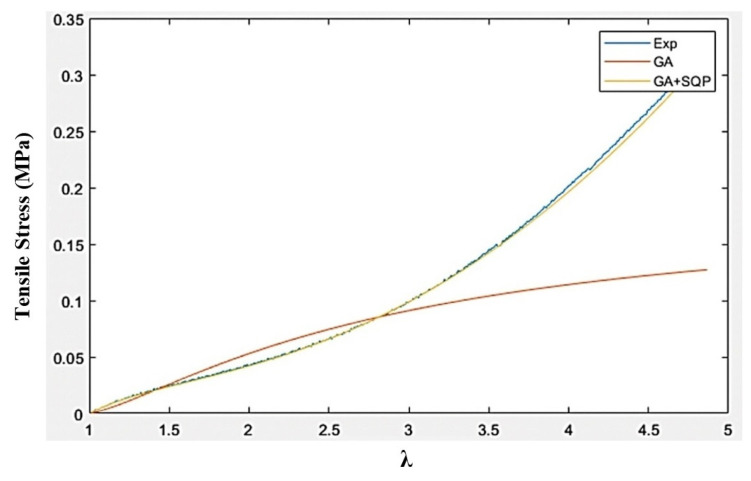
Curve-fitted plots for silicone rubber Ecoflex 30 using least-square method using GA and hybrid GA + SQP methods.

**Figure 6:**
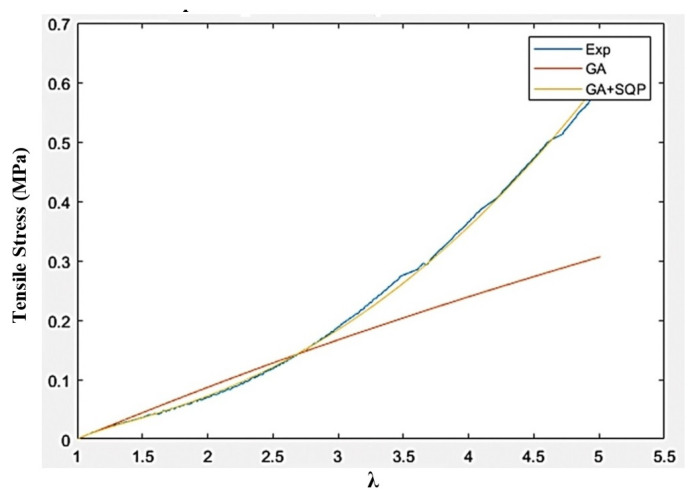
Curve-fitted plots for silicone rubber Ecoflex 50 using least-square method using GA and hybrid GA + SQP methods.

Figure 5 and Figure 6, respectively, show the comparison of stress–stretch response of silicone rubber Ecoflex 30 and Ecoflex 50 samples extracted from experiments with those obtained using the Ogden model based on optimal material parameters identified using GA and GA + SQP. Results clearly show that Ogden material model, with optimal material parameters identified through GA + SQP, provides reasonable agreement with the experimental data.

#### 2.6.2. Magnetic Properties of Carbonyl Iron Particles

**Figure 7:**
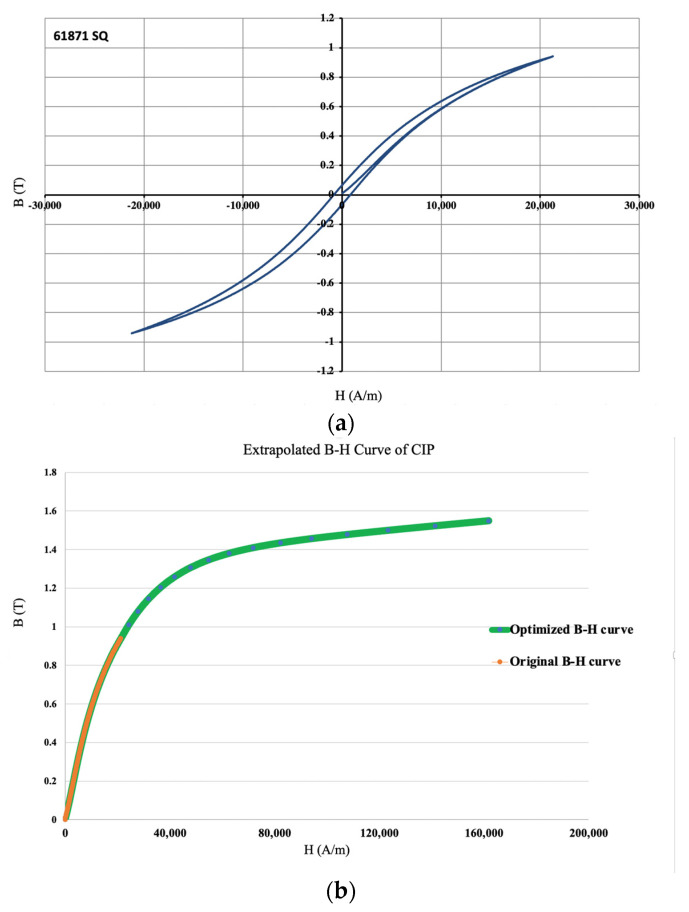
(**a**) B-H curve for CIP, provided by the manufacturer (BASF SE, Ludwigshafen, Germany), and (**b**) extrapolated B-H curve of CIP. Vertical axis represent magnetic flux density (B) in Tesla, and the horizontal axis is the field intensity in A/m.

The magnetic properties of CIPs, in the form of hysteresis *B-H* curve, were provided by the manufacturer, BASF SE, Ludwigshafen, Germany, as depicted in Figure 7a. Using the experimental *B-H* data, the following equation can be effectively used to predict *B-H* response of CIPs up to saturation [49].

(31) $$B\left(H\right)=B_{s}\left(1-ae^{-bH}\right)+\mu_{0}H$$

In which, *B* and *H* are magnetic flux density and magnetic field intensity, respectively. $B_{s}\;$ is the magnetic flux density at saturation, *a* and *b* are unknown magnetic parameters, and $\mu_{0}=4\pi \times 10^{-7}$ $\left[\frac{N}{A^{2}}\right]$ is the vacuum permeability. Using Equation (31), the following *B-H* curve, shown in Figure 7b, was interpolated and extrapolated for CIP, using the provided COMSOL Multiphysics plug-in.

The identified material properties to be considered in the modeling, along with the optimized parameters for interpolation and extrapolation conducted on the *B-H* curve of CIP, using Equation (31) are provided in Table 3.

## 3. Results

### 3.1. Modeling the 2D Isotropic MRE-RVE in COMSOL

The 2D MRE-RVE was generated in COMSOL using a simple cube containing one CIP inclusion. The mechanical and magnetic data associated with each part (matrix, inclusion, and the surrounding air domain) were defined precisely, according to the previous section. In order to validate the model, the first modeling was conducted on silicone rubber Ecoflex 50 with 15% volume fraction of CIP to compare with the experimental results from the literature [21].

**Figure 8:**
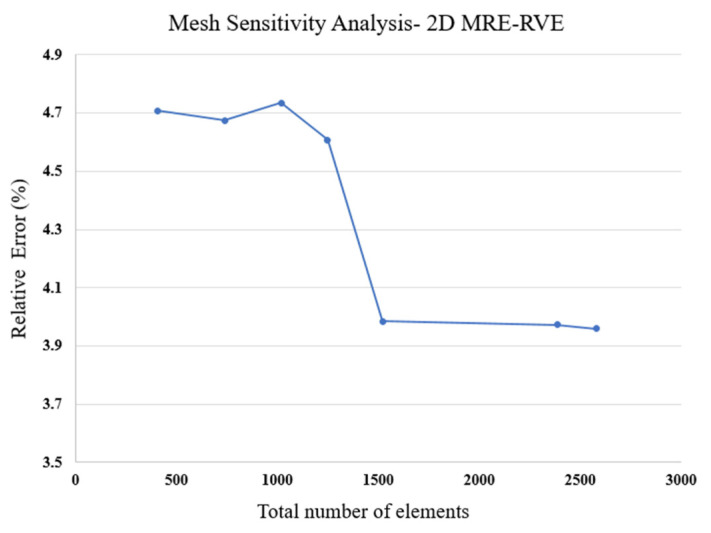
Mesh sensitivity analysis graph for 2D MRE-RVE model, Ecoflex 50 (ϕ = 15%, B = 0.2 T).

As for the meshing pattern, a user-defined mesh approach was employed to discretize the matrix, CIP, and the surrounding air. This methodology ensures precise control over meshing details, allowing for a finer mesh size in specific regions, such as boundaries, and coarsening where needed, especially within the air domain. A mesh sensitivity analysis was performed to determine the most efficient number of elements, balancing computational cost with the attainment of reasonable results. Results for mesh sensitivity for 2D MRE-RVE with 15% volume fraction of CIP and exposed to magnetic flux density of 0.2 T is provided for Ecoflex 50, in Figure 8 as an example. A relative error between the shear modulus obtained from the MRE-RVE modeling and the experimental results [21] is then defined as follows: $\frac{G_{MRE-RVE}-G_{Exp}}{G_{Exp}}\times 100.\;$It can be observed that the decrease in the relative error is negligible when the number of elements exceeds 1500.

**Figure 9:**
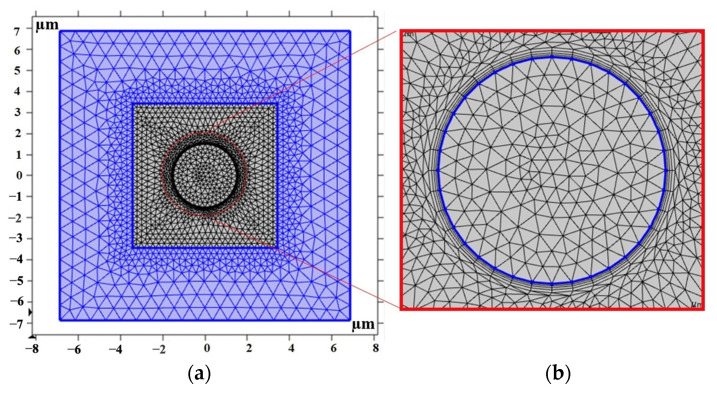
(**a**) The mesh pattern of MRE-RVE (the grey square containing the circle) surrounded by the air domain (the purple square) and (**b**) the boundary layers (the grey discretized circles) implemented to enhance precision around the inclusion (the blue circle). The cut-out section is magnified by the scale of 3.5.

Figure 9 shows the FE model of the 2D MRE-RVE. As depicted in Figure 9a and as previously discussed, the mesh employed in the air domain gets coarser as it recedes the RVE boundaries. This is considered as the minimal mechanical or magnetic loading and displacements expected within this region. In contrast, the mesh is finely dispersed around the inclusion, using four boundary layers to ensure the necessary precision in that region. This is essential due to the concentrated interaction of magnetic and mechanical forces within this area. Figure 9b illustrates the boundary layers surrounding the inclusion. It is noteworthy that a total number of 3348 triangular elements were used to discretize the entire MRE-RVE including the surrounding air domain.

#### Shear Deformation of Isotropic MRE-RVE

**Figure 10:**
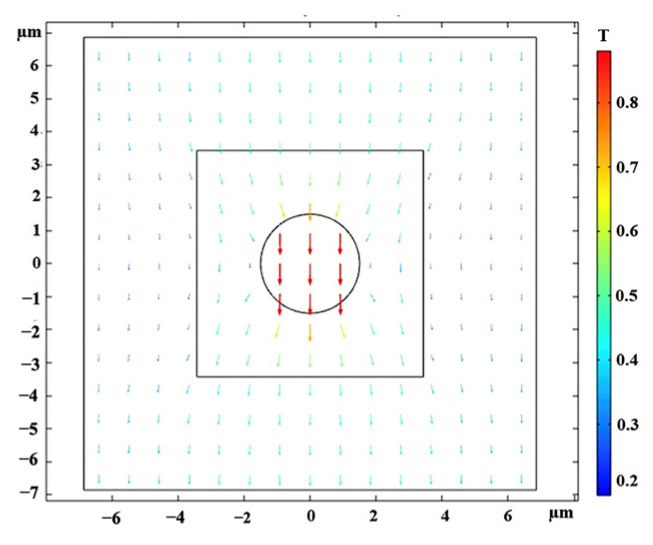
The magnetic field distortion around the CIP inclusion (the circle), while the small square indicates the RVE boundaries, and the big square is the air domain boundary. The color definition bar depicts the magnetic flux density in Tesla.

**Figure 11:**
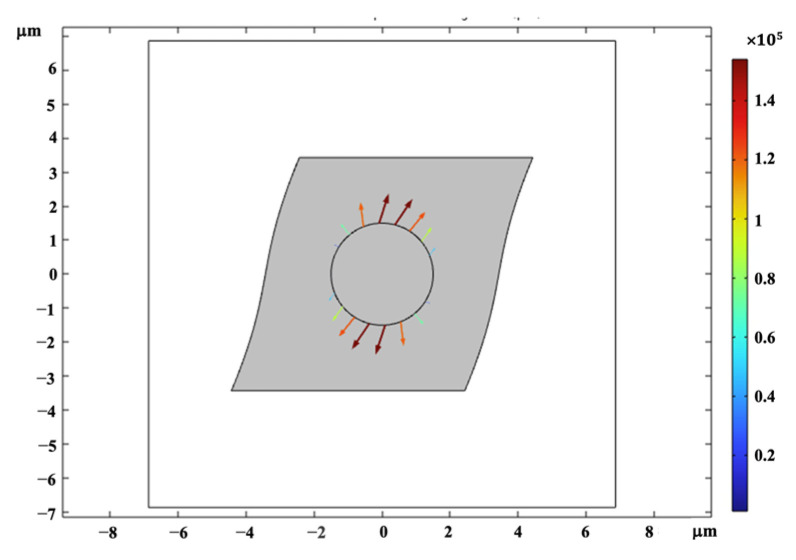
The Maxwell stress distribution generated at the CIP boundaries under magnetic flux density of 0.4 T, at 30% shear strain. The color definition bar depicts the Maxwell stress in Pa.

**Figure 12:**
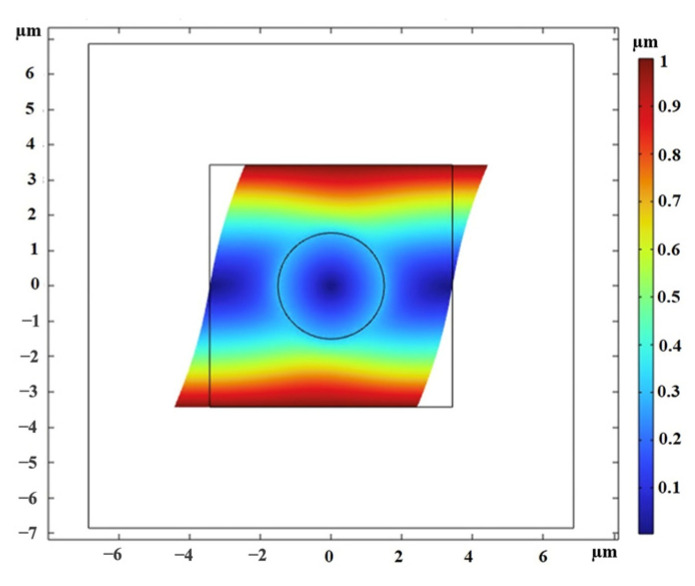
The shear deformation of MRE-RVE under 30% shear strain, while the periodic boundary conditions are applied on the RVE boundaries. The small square frame shows the RVE boundaries before deformation and the deformed RVE is colored. The color definition bar depicts the displacement magnitude in μm.

Once the MRE-RVE is constructed, a shear deformation is incrementally applied on the top face of RVE up to 30% shear strain, while the periodic boundary conditions are enforced on the edges. The shear deformation is conducted under magnetic field flux densities ranging from 0 to 0.7 T, applied perpendicular to the shear direction. Figure 10 provides an illustration of the applied magnetic field on the RVE and the distortion of the magnetic field around CIP inclusion, as being absorbed by the inclusion. The induced uniform magnetic flux density inside the inclusion is also obvious in this figure. Maxwell stress tensor is applied on the inclusion boundaries and in combination with the mechanical stress, the total shear stress generated in the RVE is calculated. Figure 11 presents the Maxwell stress distribution at the CIP boundaries, under a magnetic field of 0.4 T, when the RVE undergoes 30% shear strain. Figure 12 shows the shear deformation experienced by MRE-RVE under 30% shear strain, while the periodic boundary conditions are being applied on the RVE boundaries. It is noted that the results are provided for the silicone rubber Ecoflex 50 MRE-RVE, with 15% volume fraction of CIP under the application of 0.4 T magnetic flux density as an example. In Figure 10, Figure 11 and Figure 12, the smaller square indicates the RVE boundaries, while the bigger one represents the air domain boundaries.

**Figure 13:**
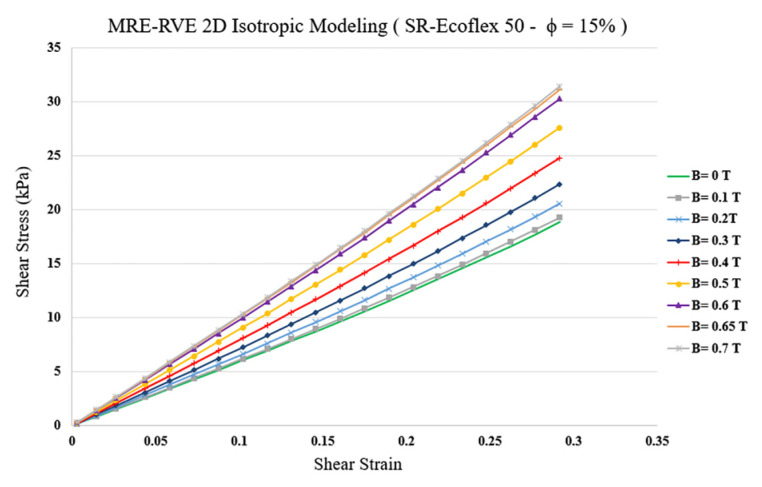
Shear stress–shear strain plot for 2D MRE-RVE under different applied magnetic fields.

**Figure 14:**
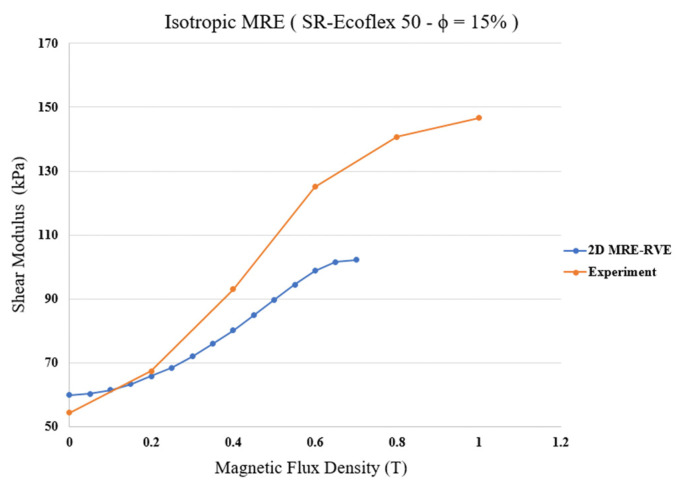
Comparison of shear modulus versus magnetic flux density obtained from COMSOL FE modeling of 2D isotropic MRE-RVE with the experimental results [21] for silicone rubber Ecoflex 50-MRE with 15% volume fraction of CIP.

Finally, the pure shear analysis was conducted to obtain shear stress–shear strain response of MRE-RVE under the application of different magnetic flux densities. Figure 13 represents the homogenized shear stress versus shear strain behavior of the silicone rubber Ecoflex 50 MRE-RVE containing 15% CIP in volume fraction, under an external magnetic field ranging from 0 to 0.7 T. Examination of results in Figure 13 reveals that shear modulus, representing the slope of the shear stress–shear strain curves, substantially increases by increasing the magnetic field intensity. For instance, at nearly 30% shear strain, the generated shear stress increases almost over 60% from nearly 19 kPa to almost 31 kPa by increasing the magnetic flux density from 0 to 0.7 T, respectively. Results also show that the change in modulus decreases as the magnetic flux density increases, confirming the saturation phenomenon. The variation in the shear modulus with respect to the magnetic flux density obtained from the 2D isotropic MRE-RVE, and its comparison with the reported experimental results is shown in Figure 14.

Results show that the zero-field shear modulus of MRE-RVE obtained from COMSOL FE modeling is 59.9 kPa, which is 10% higher than the 54.43 kPa zero-field shear modulus of MRE given by experimental results. Results from 2D MRE-RVE model substantially deviates from experiential results as magnetic flux density increases beyond 0.2 T. Moreover, Figure 14 illustrates that the field-induced shear modulus of MRE-RVE reaches saturation at magnetic flux density of nearly 0.65 T as also evident from Figure 13, while that of the experiment keeps increasing up to 0.8 T of applied magnetic field. The differences between modeling and experimental results are quantified in Table 4.

Results show that while 2D isotropic MRE-RVE model may provide acceptable results for shear modulus results under lower magnetic field, it cannot capture the magneto-mechanical behavior of MREs under higher magnetic fields. For example, the differences between shear moduli from 2D FE modeling and experiment are 10% at 0 T, −11% at 0.4 T, and −20% at 0.7 T.

**Figure 15:**
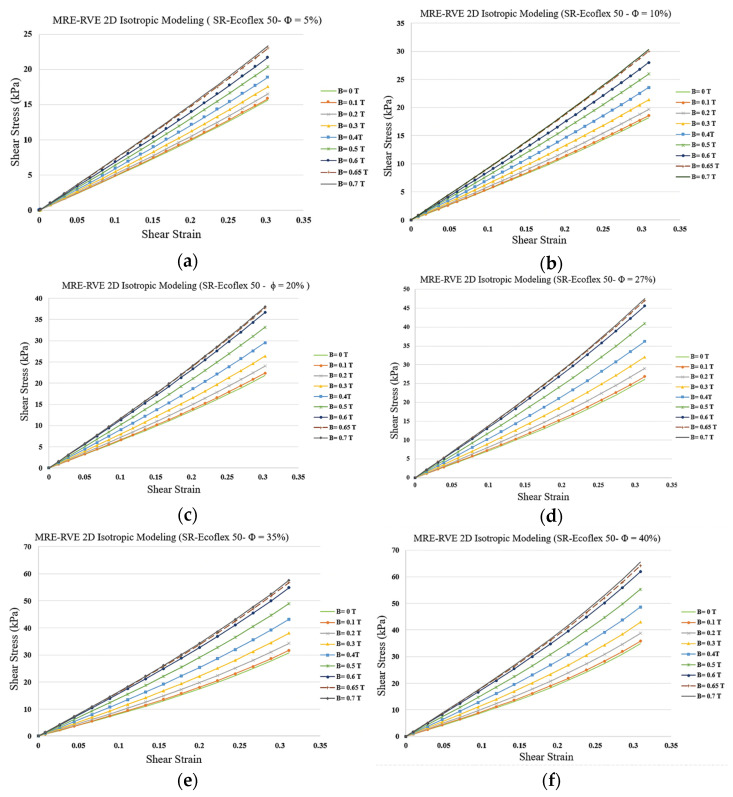
Shear stress–shear strain plot for 2D MRE-RVE under different applied magnetic fields for silicone rubber (SR) Ecoflex 50 with (**a**) 5%, (**b**) 10%, (**c**) 20%, (**d**) 27%, (**e**) 35%, and (**f**) 40% of CIP in volume fraction.

The developed 2D isotropic MRE-RVE FE model was subsequently used to qualitatively investigate the effect of CIP volume fraction on the shear modulus. Figure 15a–f show the results for the shear stress–shear strain response behavior of MREs under different magnetic flux densities for CIP volume fraction ranging from 5% to 40%. Results show that increasing the volume fraction of CIP yields higher field-induced shear modulus. For instance, when increasing the magnetic flux density from 0 to 0.7 T, and under shear strain of 30%, the shear stress increases from nearly 16 kPa to almost 23 kPa (44%) and from 35 kPa to 65 kPa (86%) for CIP volume fraction of 5% and 40%, respectively.

**Figure 16:**
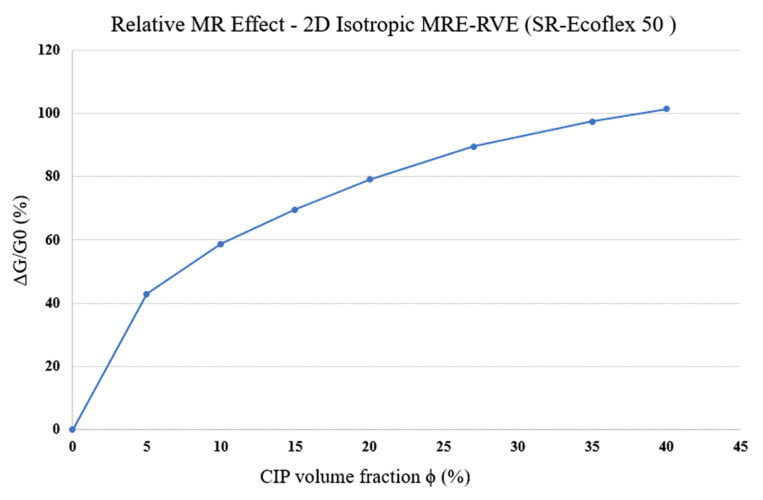
MR effect behavior in 2D modeling of silicone rubber (SR) Ecoflex 50 MRE-RVE with respect to CIP volume fraction.

As the CIP volume fraction increases, the gap between two subsequent curves in each figure (Figure 15a–f) increases, implying that the influence of magnetic field on shear modulus, and consequently, the MR effect increase as the volume fraction increases. The variation in MR effect with respect to CIP volume fraction is shown in Figure 16. Results suggest that the MR effect increases by increasing the CIP volume fraction. Although the MR effect is supposed to reach a maximum at around ϕ = 27% and then drop [17], the 2D modeling is thus not able to capture this behavior and the MR effect keeps increasing as the volume fraction goes up.

**Figure 17:**
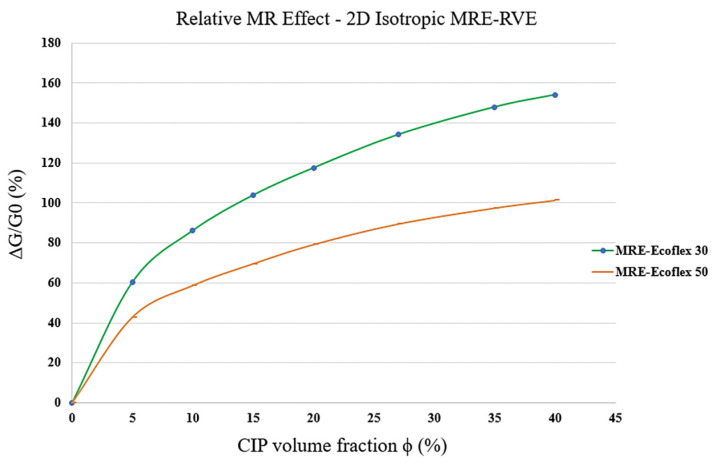
Comparison of the MR effect behavior in 2D isotropic MRE-RVEs with different matrix materials (Ecoflex 50 and Ecoflex 30) with respect to CIP volume fraction.

The same procedure of FE modeling used for silicone rubber Ecoflex 50 MRE-RVE, was also conducted on silicone rubber Ecoflex 30 MRE-RVE. The influence of different magnetic flux densities, ranging from 0–0.7 T, was also studied on the shear stress–shear strain response of the silicone rubber Ecoflex 30 MRE-RVEs, containing various CIP content. Figure 17 shows the MR effect with respect to CIP volume fraction for 2D MRE-RVE with different matrix materials (Ecoflex 30 and Ecoflex 50). Results clearly show that MR effect in the MRE-RVE with the softer matrix (Ecoflex 30) reaches a maximum of 154%, while that in Ecoflex 50 MRE-RVE reaches a maximum of 100%, both containing 40% CIP in volume fraction. Although showing a higher relative MR effect in MREs with softer matrix is anticipated due to the experimental data in the literature [21,22], the relative MR effect is supposed to reach a peak at the optimum CIP volume fraction, and then decrease as the volume fraction goes up [17], and the 2D MRE-RVE modeling cannot capture this behavior.

The difference in the results could be attributed to the incapability of 2D model to capture the whole physical phenomenon. It is noted that, in 2D RVE model, an extruded depth should be assigned to the plane geometry. Thus, the inclusion is in fact considered as a short cylindrical fiber which is different from the geometry of the nearly spherical inclusion in reality.

### 3.2. Modeling the 3D Isotropic MRE-RVE in COMSOL

As suggested in the previous section, the 2D MRE-RVE was not able to properly capture the coupled magneto-mechanical response of MREs in shear deformation. Hence, the modeling approach has been extended to 3D. Therefore, the 3D MRE-RVE was generated in COMSOL in the same fashion as that of 2D modeling. One CIP inclusion was generated and placed inside a simple cube of matrix material and surrounded by a larger cube of air. The mechanical and magnetic data associated with each part (matrix, magnetic particle inclusion, and the surrounding air domain) was also defined precisely, as explained before. To validate the model, we initiated the modeling process for silicone rubber Ecoflex 50 containing 15% volume fraction of CIP. Subsequently, we conducted a comparison with the experimental data reported in the literature [21].

In order to create a complex and customized structure, the RVE underwent a detailed meshing procedure. We decided to utilize the “user-defined mesh” method, similar to the approach used in the 2D configuration. A tetrahedron mesh type is used, as it provides more flexibility for meshing the curved boundaries, here, the spherical magnetic particle.

**Figure 18:**
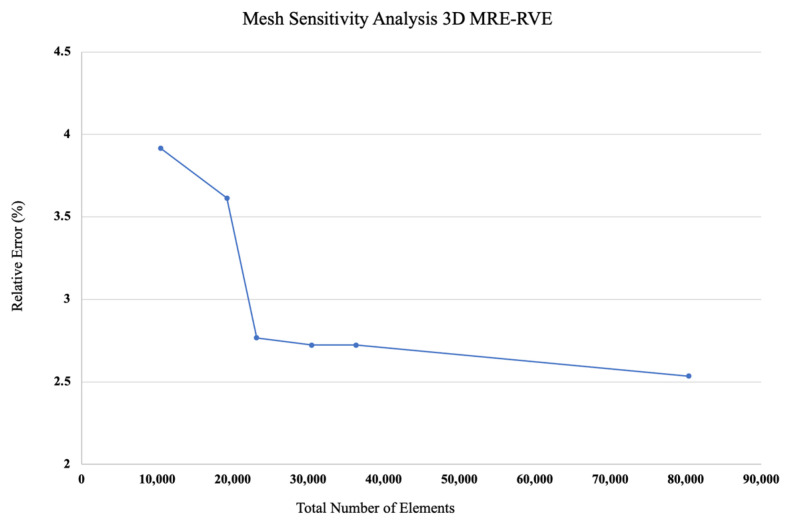
Mesh sensitivity analysis graph for 3D MRE-RVE model, (ϕ = 15%, B = 0.1 T) using different meshing schemes.

A methodical mesh sensitivity analysis was then systematically performed to reach the optimal mesh pattern, ensuring that computational resources were not needlessly burdened. Results of the relative error between the shear modulus obtained from the 3D MRE-RVE modeling and the experimental results [21], defined as: $\frac{G_{MRE-RVE}-G_{exp}}{G_{exp}}\times 100$, for different number of elements are provided in Figure 18. Just as described in the 2D modeling section, we explored various meshing configurations while creating the 3D model. Results in Figure 18 show that the relative error between the shear modulus obtained from 3D MRE-RVE and experiments decreases as the total number of tetrahedron elements increases, indicating the convergence of the shear modulus. Hence, based on this finding and by evaluating the computational cost, we opted for the mesh configuration consisting of 30,492 tetrahedral elements to balance between the computational cost and accuracy.

**Figure 19:**
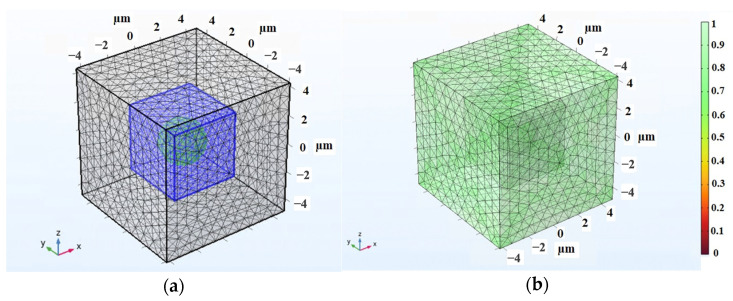
(**a**) The mesh pattern of MRE-RVE (the blue cubic matrix containing the green spherical inclusion) surrounded by the air domain (the grey cube) and (**b**) the mesh quality in all regions with the color bar representing the quality of mesh on the scale of 0 to 1.

A visual representation of the mesh pattern applied in the 3D MRE-RVE modeling is provided in Figure 19a. As previously explained, the mesh density in the air domain progressively coarsens as it moves away from the RVE boundaries, for the anticipation of minimal mechanical or magnetic loading and displacements in this particular zone. Conversely, the mesh is finely adjusted in the vicinity of the inclusion, to ensure the required precision in that area due to the intensified interaction of magnetic and mechanical forces. Figure 19b further illustrates the mesh quality in all regions.

**Figure 20:**
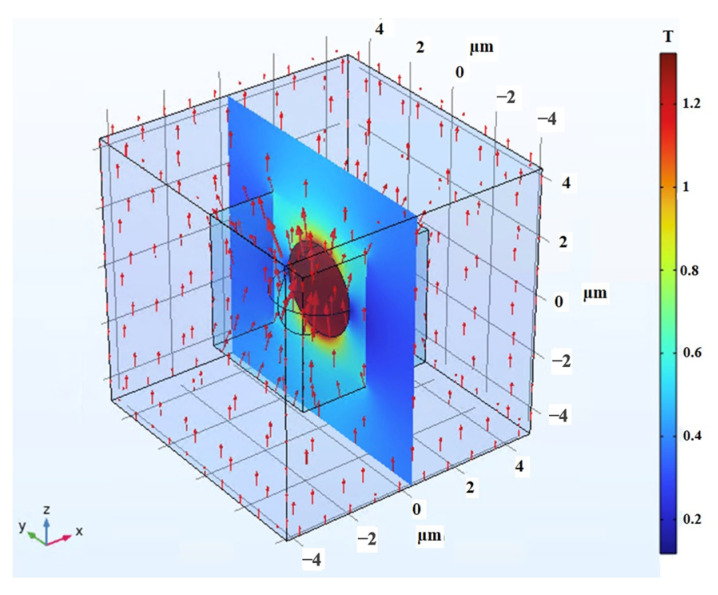
The magnetic field distortion around the CIP inclusion inside the 3D RVE, red arrows represent the magnetic field intensity and direction (0.1 T, upward), the color definition bar describes the magnetic flux density (T) in the air, and MRE-RVE domain, referring to the hypothetical cut out surface in the middle of the model.

**Figure 21:**
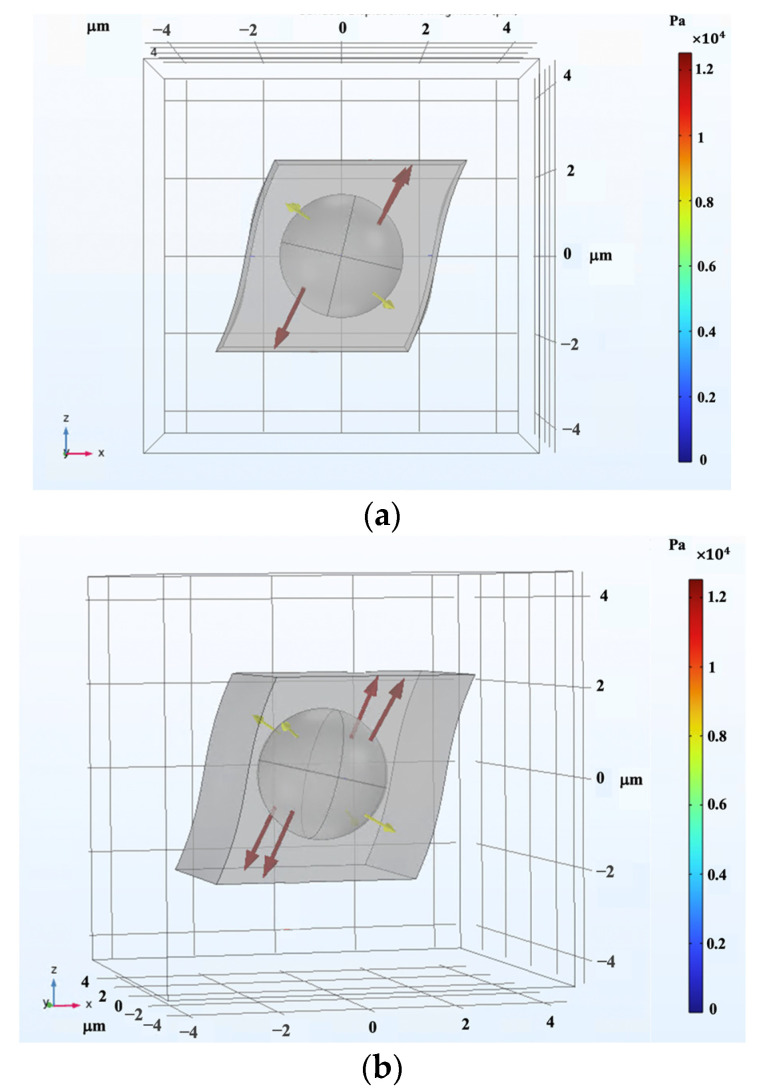
The Maxwell stress distribution generated at the CIP boundaries under magnetic flux density of 0.1 T, at 30% shear strain: (**a**) x–z plane view and (**b**) spatial 3D view. The arrows represent the Maxwell stress, and the color definition bar depicts the Maxwell stress in Pa.

As for shear analysis, the RVE was systematically subjected to incremental pure shear deformation, gradually reaching a shear strain of 30%. To maintain consistency, the periodic boundary conditions were imposed along all surface boundaries. Concurrently, a magnetic field was applied perpendicular to the shear direction, spanning a range of magnitudes from 0 to 0.4 T. The visual representation in Figure 20 clearly portrays the magnetic field’s interaction with the RVE, particularly highlighting the distortion of the field as it encounters the CIP inclusion. The high induced magnetic flux density within the inclusion is clearly visible. In the same fashion, with the 2D modeling, the Maxwell stress tensor was applied to the boundaries of the CIP inclusion, calculated at each step of the analysis, and simultaneously interpreted as a mechanical load into the modeling in solid mechanics physics, interpreting the whole coupled problem as a solid mechanic problem with periodic boundary conditions applied accordingly. Integrating this stress with the mechanical stress developed by the shear deformation, the overall shear stress generated within the 3D MRE-RVE was determined. Figure 21a,b illustrate the Maxwell stress distribution at the CIP boundaries under a magnetic field of 0.1 T, when the RVE undergoes 30% shear strain.

**Figure 22:**
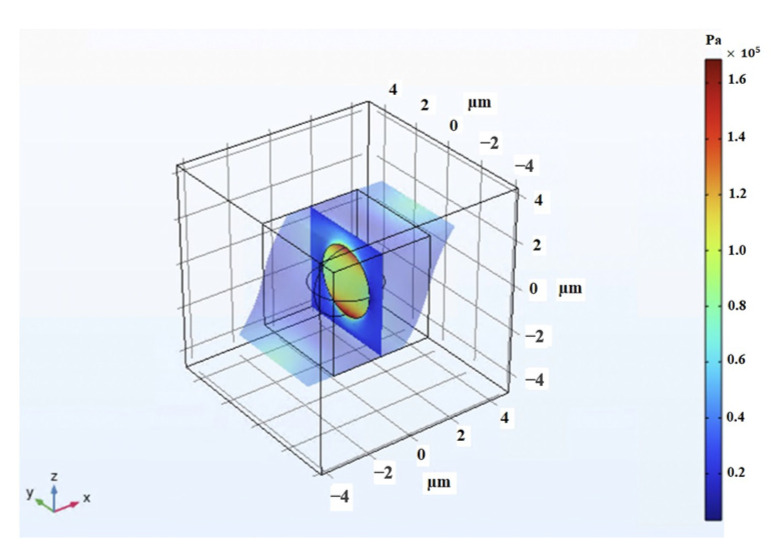
The shear deformation of MRE-RVE under 30% shear strain, while the periodic boundary conditions are being applied on the RVE boundaries, subjected to a magnetic field of 0.1 T. The smaller cubic frame shows the RVE boundaries before deformation and the deformed RVE is colored. The color definition bar depicts the Tresca stress in Pa.

Figure 22 illustrates a visualization of the shear deformation of the MRE-RVE under shear strain of 30% and the shear stress distribution throughout the entire MRE-RVE. The RVE boundaries are consistently depicted by the smaller cube, while the larger cube delineates the boundaries of the air domain. It is worth noting that Figure 20, Figure 21 and Figure 22 feature the silicone rubber Ecoflex 50 MRE-RVE with a 15% volume fraction of CIP, subjected to a magnetic field of 0.1 T.

#### 3.2.1. MRE-RVE-Silicone Rubber Ecoflex 50

**Figure 23:**
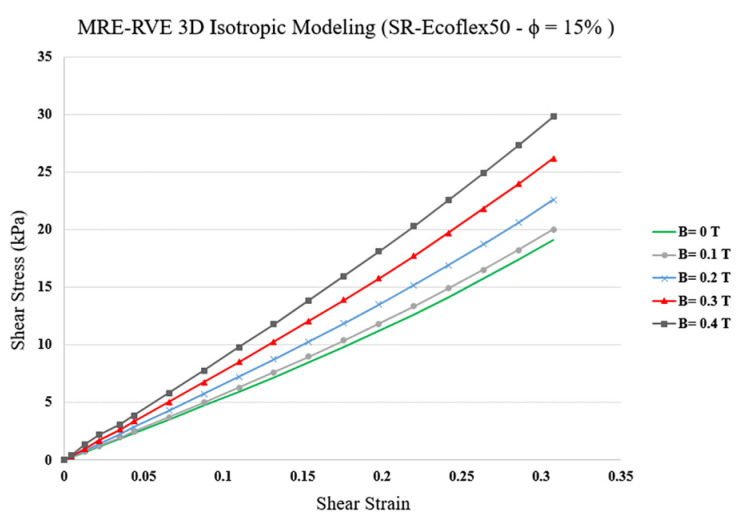
Shear stress–shear strain plot for 3D MRE-RVE (silicone rubber (SR) Ecoflex 50), containing 15% of CIP in volume fraction under different applied magnetic fields.

The pure shear analysis was subsequently conducted on MRE-RVE with the silicone rubber Ecoflex 50 as the matrix material under the application of magnetic flux densities ranging from 0 to 0.4 T. Results for the homogenized shear stress versus shear strain for MRE-RVE containing 15% CIP in volume fraction under the application of varied magnetic flux densities are shown in Figure 23.

**Figure 24:**
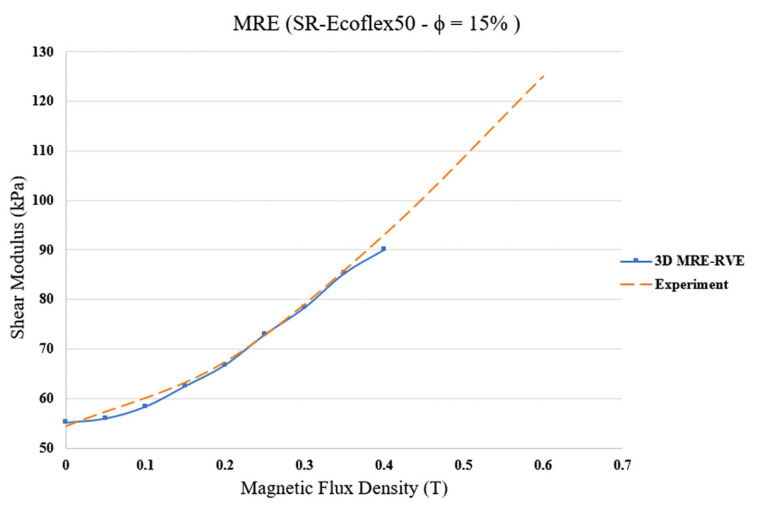
Shear modulus versus magnetic flux density for 3D isotropic MRE-RVE (silicone rubber (SR) Ecoflex 50).

Examination of the results in Figure 23 reveals that, as expected, the shear modulus increases by increasing the magnetic field intensity. In other words, as the magnetic field increases, the MRE-RVE experiences higher shear stress while undergoing the same amount of shear strain. For example, when undergoing 30% shear strain, the MRE experiences roughly 18 kPa of shear stress when no magnetic field is applied; however, by increasing the magnetic field up to 0.4 T, the shear stress reaches almost 30 kPa, indicating a 66% increase. The variation in the predicted field-dependent shear modulus with respect to the applied magnetic flux density and its comparison with reported experimental results [21] are shown in Figure 24. It is observed that, unlike the 2D MRE-RVE model, the 3D MRE-RVE can accurately predict the field-dependent shear modulus of the MRE up to 0.4 T. For instance, the zero-field shear modulus of MRE-RVE obtained from COMSOL FE 3D modeling is 55.15 kPa, which is only 1.3% higher than the 54.43 kPa zero-field shear modulus of MRE obtained experimentally.

In order to assure that the whole behavior of the MRE is captured accurately in the FE modeling, the coefficient of determination ($R^{2}$) is determined, which is defined as $R^{2}=1-\frac{SS_{res}}{SS_{tot}}$. $SS_{res}$ represents the sum of squared residuals (the differences between the predicted values and the actual values), and $SS_{tot}$ is the total sum of squares, which measures the total variance of the predicted variable. $R^{2}$ close to one describes a perfect agreement between the predicted values and the actual values. $R^{2}$ was determined between the results from the modeling and the ones obtained from experiments [21], and is found to be 0.99.

We have attempted to evaluate the shear response behavior of the 3D-RVE modeling for higher magnetic flux densities, beyond 0.4 T. However, the model fails due to the complex interaction between the mechanical and magnetic loads. Analyzing the results, we realized that the issue is likely due to the abrupt change in the material properties between an extremely soft rubber and a rigid inclusion, along with the accumulated mechanical and magnetic nonlinearity associated with the stress and material behavior.

**Figure 25:**
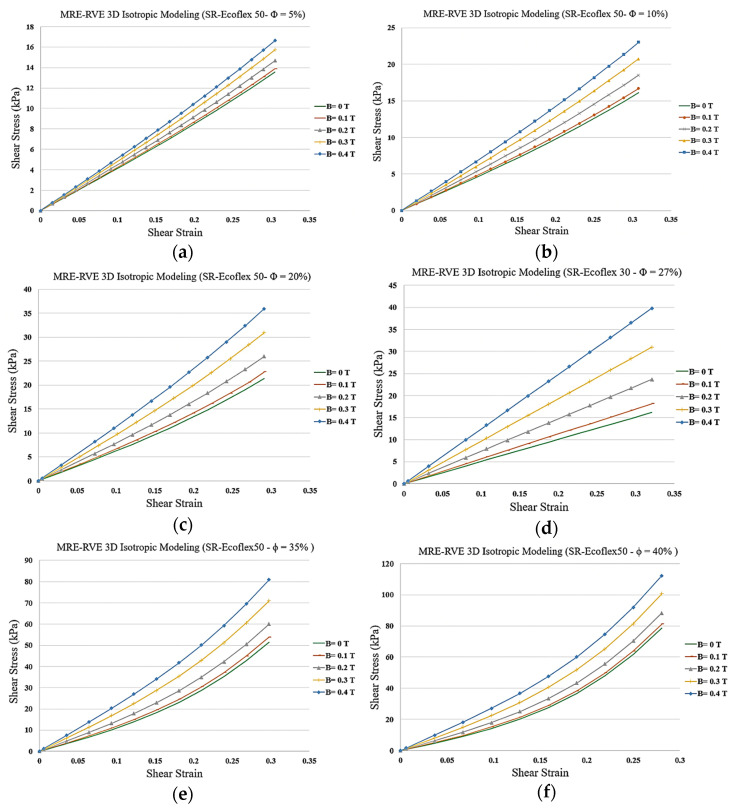
Shear stress–shear strain plot for 3D isotropic MRE-RVE under different applied magnetic fields for silicone rubber (SR) Ecoflex 50 with (**a**) 5%, (**b**) 10%, (**c**) 20%, (**d**) 27%, (**e**) 35%, and (**f**) 40% of CIP in volume fraction.

The 3D MRE-RVE model was then effectively utilized to investigate the influence of CIP volume fraction, magnetic field, and matrix stiffness on the shear deformation response behavior of MREs, under varying magnetic flux densities. Results for shear stress-shear strain response of MREs with matrix Ecoflex 50 concerning different CIP volume fraction, ranging from 5% to 40%, are illustrated in Figure 25a–f.

As suggested by the results in Figure 25a–f, the nonlinearity in the stress–strain curves increases by increasing the volume fraction of CIP and by increasing the applied magnetic field. Results also show that increasing the volume fraction of CIP yields a substantial increase in the magnetic field induced shear stress at the given shear strain. For instance, at 30% shear strain and under a magnetic flux density of 0.4 T, the MRE-RVE containing 5% CIP experiences a shear stress of roughly 17 kPa, while the shear stress reaches 110 kPa for MRE-RVE containing 40% CIP. From a broader perspective, as the CIP volume fraction increases, the gap between the stress–strain curves increase, indicating an enhancement in the relative MR effect. However, the most widened gaps are observed in MRE-RVE with 27% CIP, indicating that the maximum relative MR effect occurs at the volume fraction of 27%.

**Figure 26:**
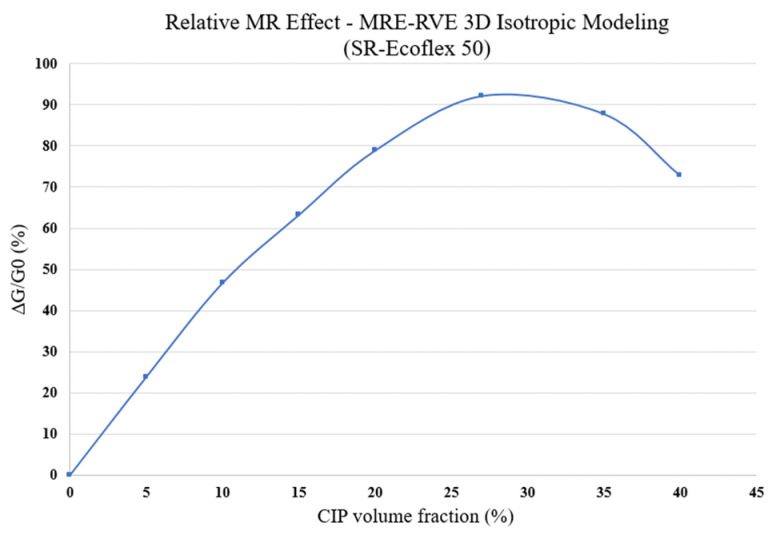
Relative MR effect for silicone rubber (SR) Ecoflex 50 MRE-RVE versus CIP volume fraction.

To have a better understanding of the influence of CIP volume fraction, the MR effect of the 3D MRE-RVEs with different volume fractions of CIP has been evaluated. It is noted that, in determining the MR effect, the maximum shear modulus was evaluated at magnetic flux density of 0.4 T. The results are shown in Figure 26, suggesting that the relative MR effect initially increases as the CIP content increases, reaching to a maximum level of 92% at 27% volume fraction as predicted before and then decreases with further increasing the volume fraction of CIP. This is in agreement with results reported by Davis [17].

#### 3.2.2. Silicone Rubber Ecoflex 30 MRE-RVE

**Figure 27:**
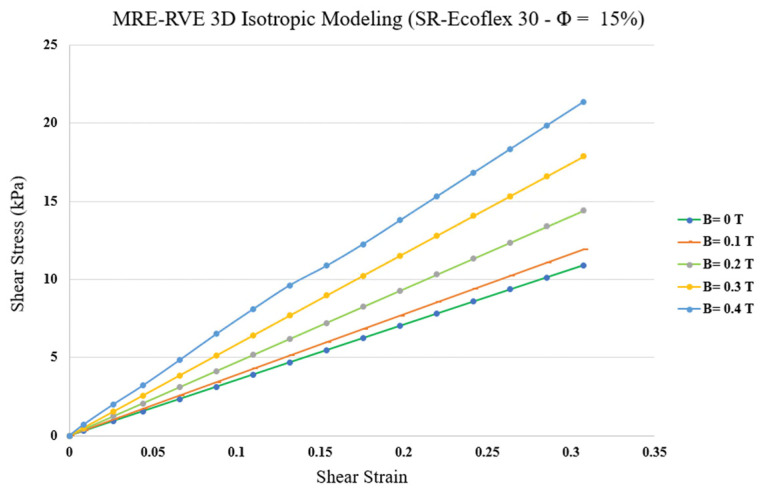
MRE-RVE (silicone rubber (SR) Ecoflex 30) shear stress–strain behavior under the application of different magnetic fields.

The same procedure of FE modeling used for silicone rubber Ecoflex 50 MRE-RVE was conducted on MRE-RVE with silicone rubber Ecoflex 30 as the matrix material. The influence of different magnetic flux densities, ranging from 0 to 0.4 T, was also studied on the shear stress–shear strain response of the silicone rubber Ecoflex 30 MRE-RVEs containing various CIP content. Figure 27 shows the shear stress–shear strain behavior of Ecoflex 30 MRE-RVEs with 15% volume fraction of CIP, under varied magnetic flux densities ranging from 0 to 0.4 T. Results in Figure 27 clearly suggest that Ecoflex 30 MRE-RVE exhibits lower stiffness and less nonlinear behavior compared with Eoflex 50 MRE-RVE in Figure 23. Moreover, a closer look at the gaps between two subsequent curves in both Figure 23 and Figure 27 reveals that, in Ecoflex 30 MRE-RVE, the stress–strain curves are more widened than those in Ecoflex 50 MRE-RVE, indicating a more pronounced relative MR effect obtained from the MRE with softer matrix material under identical conditions.

**Figure 28:**
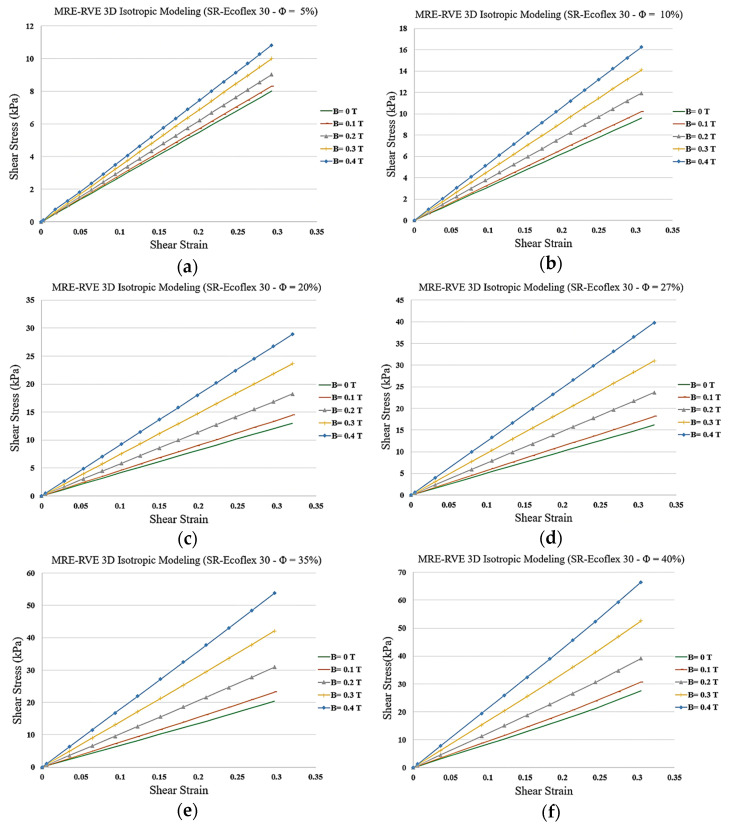
Shear stress–shear strain plot for 3D isotropic MRE-RVE under different applied magnetic fields for silicone rubber (SR) Ecoflex 30 with (**a**) 5%, (**b**) 10%, (**c**) 20%, (**d**) 27%, (**e**) 35%, and (**f**) 40% of CIP in volume fraction.

**Figure 29:**
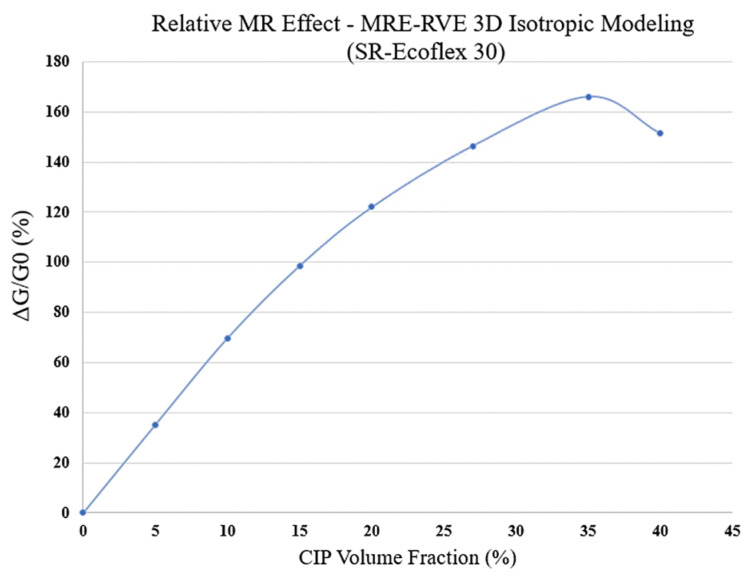
Relative MR effect versus CIP volume fraction obtained from 3D isotropic MRE-RVE for silicone rubber (SR) Ecoflex 30.

**Figure 30:**
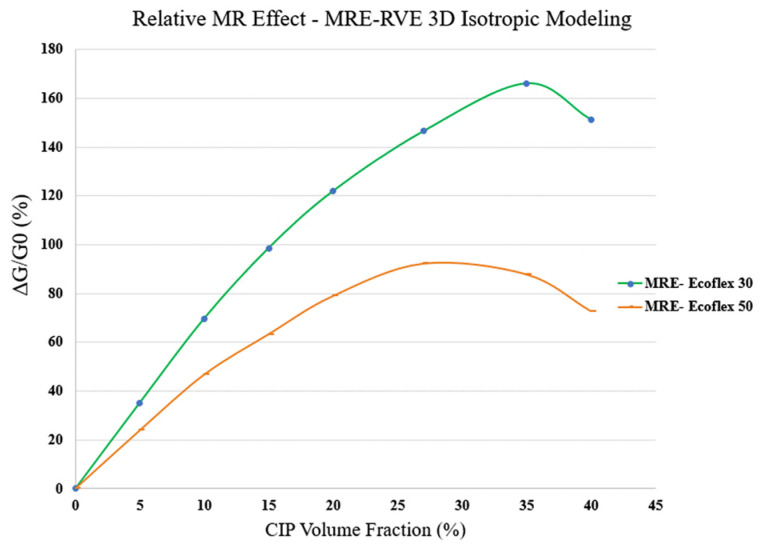
Comparing MR effect versus CIP volume fraction obtained from 3D isotropic MRE-RVE for silicone rubber Ecoflex 30 and silicone rubber Ecoflex 50.

Similar to Ecoflex 50 MRE-RVE, the effect of volume fraction of CIP on the shear stress–shear strain response of Ecoflex 30 MRE-RVE has also been investigated, and results are shown in Figure 28a–f. Results suggest an escalation in the shear modulus and the relative MR effect when the CIP content goes up. The effect of volume fraction of CIP on the relative MR effect can be better understood in Figure 29. Results show that the relative MR effect for Ecoflex 30 MRE-RVE reaches its peak at CIP volume fraction of nearly 35%, while the Ecoflex 50 MRE-RVE experienced its maximum relative MR effect at 27% CIP content (Figure 26). Figure 30 illustrates the comparison of the results for MR effect with respect to CIP content for MRE-RVE with Ecoflex 50 and Ecoflex 30 as the host matrices. Results show that the maximum MR effect obtained from the softer matrix (Ecoflex 30) was observed to be nearly 166% at 35% volume fraction of CIP compared with nearly 92% MR effect at 27% volume fraction CIP for silicone rubber Ecoflex 50. It can be observed that the maximum relative MR effect in MREs with the softer matrix is noticeably higher than that of MREs with silicone rubber Ecoflex 50, which has also been confirmed by other studies [21,22].

## 4. Conclusions

In this study, an FE model based on the representative volume element (RVE) approach was proposed to model the shear deformation of MREs under the influence of an external magnetic field, while taking all the nonlinearities into account. Experiments were carried out on host rubber samples (silicone rubber) to formulate the highly nonlinear Ogden strain energy to describe hyper-elastic behavior of the rubbery matrix. The magnetic behavior of CIP was described through a nonlinear B-H curve. The appropriate RVE size for modeling the MREs was defined, and the MRE-RVE was generated in COMSOL Multiphysics in 2D and 3D configurations. The MRE-RVE underwent incremental pure shear deformation, while periodic boundary conditions (PBCs) were imposed on the RVE boundaries. Simultaneously, a homogeneous magnetic field was applied perpendicular to the shear direction, and the Maxwell stress tensor was defined on the CIP inclusion. The study focused on isotropic MREs, investigating the influence of varied magnetic flux densities, CIP content, and the host rubber’s hyper-elastic behavior on the shear modulus of the MREs. The MR effect behavior of the MRE-RVEs was also studied and compared.

The analysis started with a comprehensive study on the 2D MRE-RVEs, generated as a simple square with one circular inclusion inside, surrounded by air. The MRE-RVE underwent incremental pure shear deformation up to 30%, and the impact of magnetic flux densities ranging from 0 to 0.7 T was investigated. The results showed that the shear modulus in the MRE-RVE keeps a positive correlation with the magnetic flux density and the CIP content. Comparing the 2D results for Ecoflex 50 with the experimental results in the literature [21] revealed that although the 2D modeling can predict the MRE’s behavior within ±20% difference with the experimental results, and shows the saturation effect, it cannot accurately predict the variation in MR effect with respect to CIP volume fraction. The relative MR effect in the 2D MRE-RVE keeps increasing by increasing the CIP volume fraction without showing any peak MR effect. These differences between the results obtained from the modeling and the experiments could be attributed to the incapability of the 2D model to capture the physical shape of the inclusions.

Subsequently, 3D MRE-RVE was developed in the COMSOL environment. The modeling was conducted on a simple cubic MRE-RVE with one spherical inclusion inside, and the whole RVE was surrounded by a larger cube of air. The study was conducted on MRE-RVEs concerning varied magnetic flux densities (ranging 0–0.4 T), CIP content, and different host elastomers (silicone rubber Ecoflex 30 and Ecoflex 50). As expected, the positive correlation between the shear modulus and the magnetic flux density as well as the CIP content was suggested by the results, in both silicone rubber based MREs. Comparing the 3D results for Ecoflex 50 with the experimental results in the literature [21] revealed that the results hold a perfect agreement with the experiments, offering a coefficient of determination ($R^{2}$) of 0.99. Exploring the MR effect in the 3D MRE-RVE showed that the relative MR effect in the MRE-RVE with the softer matrix material (Ecoflex 30) is higher than that of Ecoflex 50 MRE-RVE. The 3D modeling was also able to predict the MR effect variation with respect to CIP volume fraction accurately. The results suggested that the relative MR effect in Ecoflex 30 MRE-RVE keeps increasing up to 35% volume fraction of CIP, peaking at 166%, while the MR effect in MRE-RVE with Ecoflex 50 reaches a maximum of 92% at 27% CIP content. Results suggest that using a softer matrix material leads to a higher relative MR effect with higher optimum volume fraction.

## Figures and Tables

**Table 1:** The optimized parameters gained through curve fitting the experimental data with the Ogden strain energy function.

Material	Optimization Method	$\mu_{1}\;$ (Pa)	$\alpha_{1}$	$\mu_{2}\;$ (Pa)	$\alpha_{2}$	$\mu_{3}\;$ (Pa)	$\alpha_{3}$	*G* (kPa)
Silicone Rubber Ecoflex 50	GA	0.0849 × $10^{6}$	1.0000	0.0005 × $10^{6}$	5.5354	−0.0001 × $10^{6}$	−1.000	43.884
	GA + SQP	0.0001 × $10^{6}$	1.0000	0.0088 × $10^{6}$	3.4927	−0.0076 × $10^{6}$	−5.3724	35.833
Silicone Rubber Ecoflex 30	GA	−6.9874 × $10^{6}$	1.6760	5.2412 × $10^{6}$	1.5601	1.8713 × $10^{6}$	1.9162	25.850
	GA + SQP	4.3891 × $10^{6}$	1.4354	−4.3380 × $10^{6}$	1.4529	0.0163 × $10^{6}$	3.1195	24.141

**Table 2:** Material properties of silicone rubber.

Material	Material Properties	Value
Silicone Rubber	Density ρ	$920\;(\mathrm{Kg}/m^{3}$)
	Poisson ratio ν	~0.5 (incompressible material)
	$\mathrm{Magnetic}\;\mathrm{Relative}\;\mathrm{Permeability}\;\mu_{r}$	2

**Table 3:** Material properties of air and CIP.

Material	Material Properties	Value
Air	$\mathrm{Relative}\;\mathrm{Permeability}\;\mu_{r}$	1
CIP	Young’s Modulus E	210 GPa
	Poisson ratioν	0.33
	Density ρ	$7870\;\mathrm{Kg}/m^{3}$
Optimized Extrapolation Parameters for CIP B-H Curve	a	6.1746
	b	6.1943 m/A
	$B_{s}$	1.38 T

**Table 4:** Comparing the results of 2D MRE-RVE and the experiments.

	Zero-Field Shear Modulus (kPa)	Maximum Shear Modulus (kPa)	Saturation Magnetic Flux Density (T)	MR-Effect (ΔGG0)
MRE-RVE	59.9	102.27	0.65	70.73%
Experiments [21]	54.43	146.59	0.8	169%

## Data Availability

The datasets presented in this article are not readily available because the data are part of an ongoing study. Requests to access the datasets should be directed to the corresponding author.

## References

[1] Carlson J.D., Jolly M. (2000). MR Fluid, foam and elastomer devices. Mechatronics.

[2] Bastola A.K., Paudel M., Li L., Li W. (2020). Recent progress of magnetorheological elastomers: A review. Smart Mater. Struct..

[3] Saber A., Sedaghati R. (2023). The Modeling of Magnetorheological Elastomers: A State-of-the-Art Review. Adv. Eng. Mater..

[4] Li Y., Li J., Li W., Du H. (2014). A state-of-the-art review on magnetorheological elastomer devices. Smart Mater. Struct..

[5] Liu Y. (2023). A review on magnetorheological elastomers. Adv. Eng. Technol. Res..

[6] Bastola A.K., Hossain M. (2020). A review on magneto-mechanical characterizations of magnetorheological elastomers. Compos. Part B Eng..

[7] Jaafar M.F., Mustapha F., Mustapha M. (2021). Review of current research progress related to magnetorheological elastomer material. J. Mater. Res. Technol..

[8] Cantera M.A., Behrooz M., Gibson R.F., Gordaninejad F. (2017). Modeling of magneto-mechanical response of magnetorheological elastomers (MRE) and MRE-based systems: A review. Smart Mater. Struct..

[9] Díez A.G., Tubio C.R., Etxebarria J.G., Lanceros-Mendez S. (2021). Magnetorheological Elastomer-Based Materials and Devices: State of the Art and Future Perspectives. Adv. Eng. Mater..

[10] Li W.H., Zhang X.Z., Du H. (2013). Magnetorheological Elastomers and Their Applications. Adv. Elastomers I.

[11] Li W., Zhang X. (2010). Research and Applications of MR Elastomers. Recent Pat. Mech. Eng..

[12] Ubaidillah, Sutrisno J., Purwanto A., Mazlan S.A. (2015). Recent progress on magnetorheological solids: Materials, fabrication, testing, and applications. Adv. Eng. Mater..

[13] Sharif U., Sun B., Hussain S., Ibrahim D.S., Adewale O.O., Ashraf S., Bashir F. (2021). Dynamic behavior of sandwich structures with magnetorheological elastomer: A review. Materials.

[14] Faizal Johari M.A., Mazlan S.A., Ubaidillah Harjana Abdul Aziz S.A., Nordin N.A., Johari N., Nazmi N. (2020). An Overview of Durability Evaluations of Elastomer-Based Magnetorheological Materials. IEEE Access.

[15] Ginder J.M., Nichols M.E., Elie L.D., Tardiff J.L. (1999). Magnetorheological elastomers: Properties and applications. Smart Structures and Materials 1999: Smart Materials Technologies.

[16] Jolly M.R., Carlson J.D., Munoz B.C., Todd A. (1996). The magnetoviscoelastic response of elastomer composites consisting of ferrus particles embedded in a polymer matrix. J. Intell. Mater. Syst. Struct..

[17] Davis L.C. (1999). Model of magnetorheological elastomers. J. Appl. Phys..

[18] Berasategi J., Salazar D., Gomez A., Gutierrez J., Sebastián M.S., Bou-Ali M., Barandiaran J.M. (2020). Anisotropic behaviour analysis of silicone/carbonyl iron particles magnetorheological elastomers. Rheol. Acta.

[19] Vatandoost H., Sedaghati R., Rakheja S., Hemmatian M. (2021). Effect of pre-strain on compression mode properties of magnetorheological elastomers. Polym. Test..

[20] Syam T.M.I., Muthalif A.G.A., Salem A.M.H., Hejazi A.A.A. (2020). 3D numerical modelling and analysis of a magnetorheological elastomer (MRE). J. Vibroeng..

[21] Asadi Khanouki M., Sedaghati R., Hemmatian M. (2019). Experimental characterization and microscale modeling of isotropic and anisotropic magnetorheological elastomers. Compos. Part B Eng..

[22] Dargahi A., Sedaghati R., Rakheja S. (2019). On the properties of magnetorheological elastomers in shear mode: Design, fabrication and characterization. Compos. Part B Eng..

[23] Sun S., Peng X., Guo Z. (2014). Study on macroscopic and microscopic mechanical behavior of magnetorheological elastomers by representative volume element approach. Adv. Condens. Matter Phys..

[24] Xu H., Ye L. (2019). Characterization and Simulation of Magnetorheological Elastomer Filled with Carbonyl Iron and NdFeB Particles under Uniaxial Tension, Compression, and Pure Shear Modes. Master’s Thesis.

[25] Kiarie W.M., Jiles D.C. Modeling of Effect of Particle Size on Macroscopic Behavior of Magnetorheological Elastomers. Proceedings of the COMSOL Conference 2020.

[26] Li R., Ma S., Jin F., Yin S., Nian C., Xin N., Yu Q. Coupled multi-physics field simulation research of magneto-rheological elastomeric magneto-shear mechanical properties based on COMSOL. In Proceeding of the International Conference on Optical Technology, Semiconductor materials, and Devicesc (OTSMD 2022).

[27] Hill R. (1963). Elastic properties of reinforced solids: Some theoretical principles. J. Mech. Phys. Solids.

[28] Hashin Z. (1983). Analysis of Composite Materials. J. Appl. Mech..

[29] Evesque P. (2005). Fluctuations, Correlation and Representative Elementary Volume (REV) in Granular Materials. Poudres Grains.

[30] El Moumen A., Kanit T., Imad A. (2021). Numerical evaluation of the representative volume element for random composites. Eur. J. Mech. A/Solids.

[31] Madi K., Forest S., Jeulin D., Boussuge M. (2006). Estimating RVE sizes for 2D/3D viscoplastic composite materials. Matériaux 2006.

[32] El Moumen A., Imad A., Kanit T., Hilali E., El Minor H. (2014). A multiscale approach and microstructure design of the elastic composite behavior reinforced with natural particles. Compos. Part B Eng..

[33] Moumen AEl Kanit T., Imad A., Minor H.E.L. (2013). Effect of overlapping inclusions on effective elastic properties of composites. Mech. Res. Commun..

[34] Kanit T., Forest S., Galliet I., Mounoury V., Jeulin D. (2003). Determination of the size of the representative volume element for random composites: Statistical and numerical approach. Int. J. Solids Struct..

[35] Jeulin D. (2012). Morphology and effective properties of multi-scale random sets: A review. Comptes Rendus Mec..

[36] Willot F., Jeulin D. (2009). Elastic behavior of composites containing Boolean random sets of inhomogeneities. Int. J. Eng. Sci..

[37] Suquet P. (1987). Elements of homogenization for inelastic solid mechanics. Lect. Notes Phys..

[38] Anthoine A. (1995). Derivation of the in-plane elastic characteristics of masonry through homogenization theory. Int. J. Solids Struct..

[39] Terada K., Hori M., Kyoya T., Kikuchi N. (2000). Simulation of the multi-scale convergence in computational homogenization approaches. Int. J. Solids Struct..

[40] Van Der Sluis O., Schreurs P.J.G., Brekelmans W.A.M., Meijer H.E.H. (2000). Overall behaviour of heterogeneous elastoviscoplastic materials: Effect of microstructural modelling. Mech. Mater..

[41] Smit R.J.M., Brekelmans W.A.M., Meijer H.E.H. (1999). Prediction of the large-strain mechanical response of heterogeneous polymer systems: Local and global deformation behaviour of a representative volume element of voided polycarbonate. J. Mech. Phys. Solids.

[42] Okereke M.I., Akpoyomare A.I. (2013). A virtual framework for prediction of full-field elastic response of unidirectional composites. Comput Mater. Sci..

[43] Davis A., Onoochin V. (2020). The Maxwell Stress Tensor and Electromagnetic Momentum. Prog. Electromagn. Res. Lett..

[44] Pao Y.-H. (1978). Electromagnetic forces in deformable continua. Mech. Today.

[45] Kim B., Lee S.B., Lee J., Cho S., Park H., Yeom S., Park S.H. (2012). A comparison among Neo-Hookean model, Mooney-Rivlin model, and Ogden model for Chloroprene rubber. Int. J. Precis. Eng. Manuf..

[46] Ogden R., Saccomandi G., Sgura I. (2004). Fitting hyperelastic models to experimental data. Comput. Mech..

[47] Bergström J. (2015). Elasticity/Hyperelasticity. Mechanics of Solid Polymers.

[48] Rackl M. Curve Fitting for Ogden, Yeoh and Polynomial Models. In Proceeding of the Scilab TEC, 7th International Scilab Users Conference.

[49] Rao D.K., Kuptsov V. (2015). Effective Use of Magnetization Data in the Design of Electric Machines with Overfluxed Regions. IEEE Trans. Magn..

